# Biochar-Based Materials from Plant Waste: Physicochemical and Structural Characteristics, Zearalenone Binding In Vitro

**DOI:** 10.3390/molecules31203717

**Published:** 2026-10-16

**Authors:** Svetlana Islamova, Julia Karaeva, Svetlana Timofeeva, Evgeny Marfin, Mikhail Marfin, Nailya Mishina, Svetlana Tanaseva, Eduard Semenov, Danil Mingaleev

**Affiliations:** 1 Institute of Power Engineering and Advanced Technologies, FRC Kazan Scientific Center, Russian Academy of Sciences, 420111 Kazan, Russia; julieenergy@list.ru (J.K.); zvezdochka198512@mail.ru (S.T.); marfin76@mail.ru (E.M.); 2 Institute of Computational Mathematics and Information Technologies, Kazan Federal University, 420008 Kazan, Russia; me.marfin@gmail.com; 3 Federal Center for Toxicological, Radiation and Biological Safety, 420075 Kazan, Russia; mishinanailyan@yandex.ru (N.M.); s-tanaseva@mail.ru (S.T.); semyonovei@bk.ru (E.S.); damin80@mail.ru (D.M.)

**Keywords:** biochar, zearalenone, sustainable adsorbents, mycotoxin, plant waste, circular economy, pyrolysis

## Abstract

Contamination of feed with zearalenone (ZEN) poses a threat to animal and human health, which drives interest in low-cost sorbents derived from plant waste. For non-activated biochars, the properties that govern ZEN binding remain not fully studied. A hypothesis was proposed stating that binding efficiency depends primarily on the amount and type of the mineral fraction rather than on porosity or degree of carbonization. Four feedstocks were pyrolyzed under identical conditions (550 °C, 10 °C/min, N_2_): *Amaranthus cruentus* (AC) and *Amaranthus retroflexus* (AR) biomass (Ca- and K-rich ash; 28–34 wt%), rice husk (RH; Si-rich ash; 44 wt%), and buckwheat husk (BH; 7 wt% ash). The resulting biochars were characterized by proximate analysis, X-ray fluorescence (XRF), Fourier-transform infrared spectroscopy (FTIR), X-ray diffraction (XRD), scanning electron microscopy (SEM), low-temperature nitrogen adsorption (BET/BJH), and iodine adsorption. ZEN-binding capacity was assessed in vitro (adsorption at a nominal pH of 2.0, desorption at pH 8.0; 37 °C; toxin-to-sorbent mass ratio of 1:1000). All biochars had a very low BET specific surface area (0.45–2.30 m^2^/g). ZEN removal efficiency decreased in the order ACB > ARB > RHB > BHB (90.5 > 77.3 > 65.8 > 10.6%), and desorption did not exceed 1.5% of the bound toxin. No statistically significant correlation between any property and ZEN removal efficiency was found for the four samples; the observed order is consistent with the hypothesis that calcite-containing Ca/K ash plays an important role, but does not conclusively prove it. All biochars were non-toxic to the ciliate *Stylonychia mytilus* (survival > 70%). ACB merits further investigation, including adsorption isotherms, kinetic studies, experiments at different pH values, and in vivo trials.

## 1. Introduction

Mycotoxins are toxic secondary metabolites produced by mold fungi (micromycetes). The term “mycotoxin” literally means “fungal toxin” or “fungal poison” [1]. Most mycotoxin-producing molds belong to three main genera: Aspergillus, Penicillium, and Fusarium [2]. The most hazardous mycotoxins found in animal feed include aflatoxin B1 (AFB1), ochratoxin A (OTA), fumonisin B1 (FB1), deoxynivalenol (DON), T-2 and HT-2 toxins (T-2 and HT-2, respectively), and zearalenone (ZEN) [3]. Mycotoxins exert a range of adverse effects on various biological systems, including microorganisms, plants, animals, and humans. They pose a threat to the feed industry worldwide, directly impacting feed safety, animal health and productivity, human health through animal-derived products, the economy, and international trade [4]. A number of measures exist for the prevention of mycotoxicoses; however, they do not always yield the desired results [5,6]. Currently, considerable attention is being directed toward the search for specialized agents—enterosorbents—capable of removing mycotoxins from the bodies of animals and humans [7].

ZEN is a non-steroidal mycotoxin produced by fungi of the genus *Fusarium*, such as *Fusarium graminearum*, *Fusarium oxysporum*, *F. equiseti*, and *Microdochium nivale* [8]. ZEN is included in the list of regulated mycotoxins; due to its lipophilic properties, it is used in many studies as a model for assessing the effectiveness of feed adsorbents [9]. Due to its high heat stability, ZEN, like other mycotoxins, is difficult to completely remove during various technological operations [10].

Natural adsorbents have attracted particular interest and demonstrated a wide range of potential applications due to the growing attention to the principles of “green” chemistry [11]. Within the framework of the same “green” concept, the principle of a circular economy, aimed at using waste as a raw material that can be converted into valuable products, is particularly important [12]. Thus, the conversion of biomass into biochar can be considered a promising option for waste management.

Biochar is often marketed as horticultural charcoal and is considered a safe product of biomass processing. However, its application has extended beyond agriculture, and emerging evidence suggests its potential benefits in livestock systems [13]. Several thermochemical conversion methods can be used to produce biochar, including pyrolysis, hydrothermal carbonization, and gasification [14]. Among these, pyrolysis is the most widely used [15]. The properties of biochar can vary considerably depending on the feedstock type, its pretreatment, and technological parameters (temperature, heating rate, residence time, etc.) [16]. The use of biochar in animal feed is an emerging trend; its ability to retain toxins, including mycotoxins, is usually attributed to its porosity and surface chemistry [17].

Prosopis farcta biochar has been reported to counteract aflatoxin B1 and improve health and productivity in quails [18]. Recent studies have confirmed that under conditions simulating gastric fluid (pH 2.0), activated coconut charcoal and pine biochar could bind over 97% of ZEN at the 0.1 ppm and 1 ppm levels [19]. Modified rice straw biochar used for immobilizing *Bacillus subtilis* 168 demonstrated a ZEN detoxification rate of over 93% under in vitro conditions, which was significantly higher than that of free cells (32.48%) [20]. A study [21] showed that non-activated and activated avocado seed biochars effectively adsorb and degrade the mycotoxin ZEN, with efficiencies of 65 ± 5% and 70 ± 5%, respectively. Research [22] showed that activated carbon was the best adsorbent, binding 100% of ZEN (at pH 3 and 7.3) at dose levels of 0.1, 0.25, 0.5, and 1%. It should be noted that studies specifically focusing on biochars for ZEN mycotoxin adsorption remain scarce at present. Moreover, mycotoxin-contaminated feedstocks can be subjected to pyrolysis for detoxification and the production of clean biochar [23]. The authors of [24] also assert that research evaluating the effects of biochar supplementation in animal feed remains insufficient.

It should be noted that the studies cited above [19,20,21,22] dealt mainly with activated carbons or chars with a high specific surface area, and the effect of their mineral composition on mycotoxin adsorption was not analyzed. In conventional biochar applications (removal of metals and organic pollutants from water), ash minerals are known to affect sorption in two ways: they can create additional binding sites, but they can also block pores and reduce the accessible surface area [25,26]. However, this question has not previously been addressed for ZEN. A hypothesis was proposed stating that for non-activated biochars with a low specific surface area, the ability to bind ZEN under conditions simulating the gastrointestinal tract is determined primarily by the amount and type of the mineral (ash) fraction rather than by porosity or degree of carbonization. To test this hypothesis, biochars with contrasting compositions were selected: low ash content (less than 10%; buckwheat husk biochar); rich in K and Ca with high ash content (more than 20% and 30%; two amaranth species biochar); rich in Si with high ash content (more than 40%; rice husk biochar). Because the number of samples is small, the hypothesis was tested by qualitative comparison, and correlation analysis was used for descriptive purposes only.

The novelty of this work is threefold. First, biochars derived from *Amaranthus cruentus*, *Amaranthus retroflexus*, and buckwheat husk have not previously been studied as ZEN adsorbents. Second, all four feedstocks were pyrolyzed under identical conditions (550 °C, 10 °C/min, N_2_), so the resulting biochars differ mainly in their feedstock-derived mineral composition. This makes it possible to compare three contrasting ash types within a single study (Ca/K-carbonate-rich, Si-rich, and low-ash); at the same time, the feedstocks also differ in their organic composition, which limits the ability to separate the effect of the mineral fraction from that of other factors. Third, ZEN-binding capacity was assessed using a two-stage pH-shift model (adsorption at pH 2.0 followed by desorption at pH 8.0), which captures both uptake efficiency and binding stability during the transition from the stomach to the intestine, combined with a bioassay for general toxicity.

The aim of this study was to determine whether the mineral fraction of non-activated biochars derived from plant waste is associated with their ability to bind ZEN under conditions simulating the gastrointestinal tract. The objectives were: (1) to produce biochars from *Amaranthus cruentus* and *Amaranthus retroflexus* biomass and from rice and buckwheat husks under identical pyrolysis conditions and to determine the mass balance; (2) to characterize the biochars by proximate analysis, X-ray fluorescence spectrometry (XRF), Fourier-transform infrared spectroscopy (FTIR), X-ray diffraction (XRD), scanning electron microscopy (SEM), low-temperature nitrogen adsorption (BET/BJH methods), and iodine adsorption; (3) to determine ZEN adsorption at pH 2.0 and desorption at pH 8.0 in vitro (37 °C; toxin-to-sorbent mass ratio of 1:1000); (4) to compare ZEN removal efficiency with ash content, ash type, porosity, and fixed carbon content; and (5) to carry out a preliminary assessment of general toxicity using the ciliate *Stylonychia mytilus*.

## 2. Results

### 2.1. Material Balance of Pyrolysis

**Figure 1:**
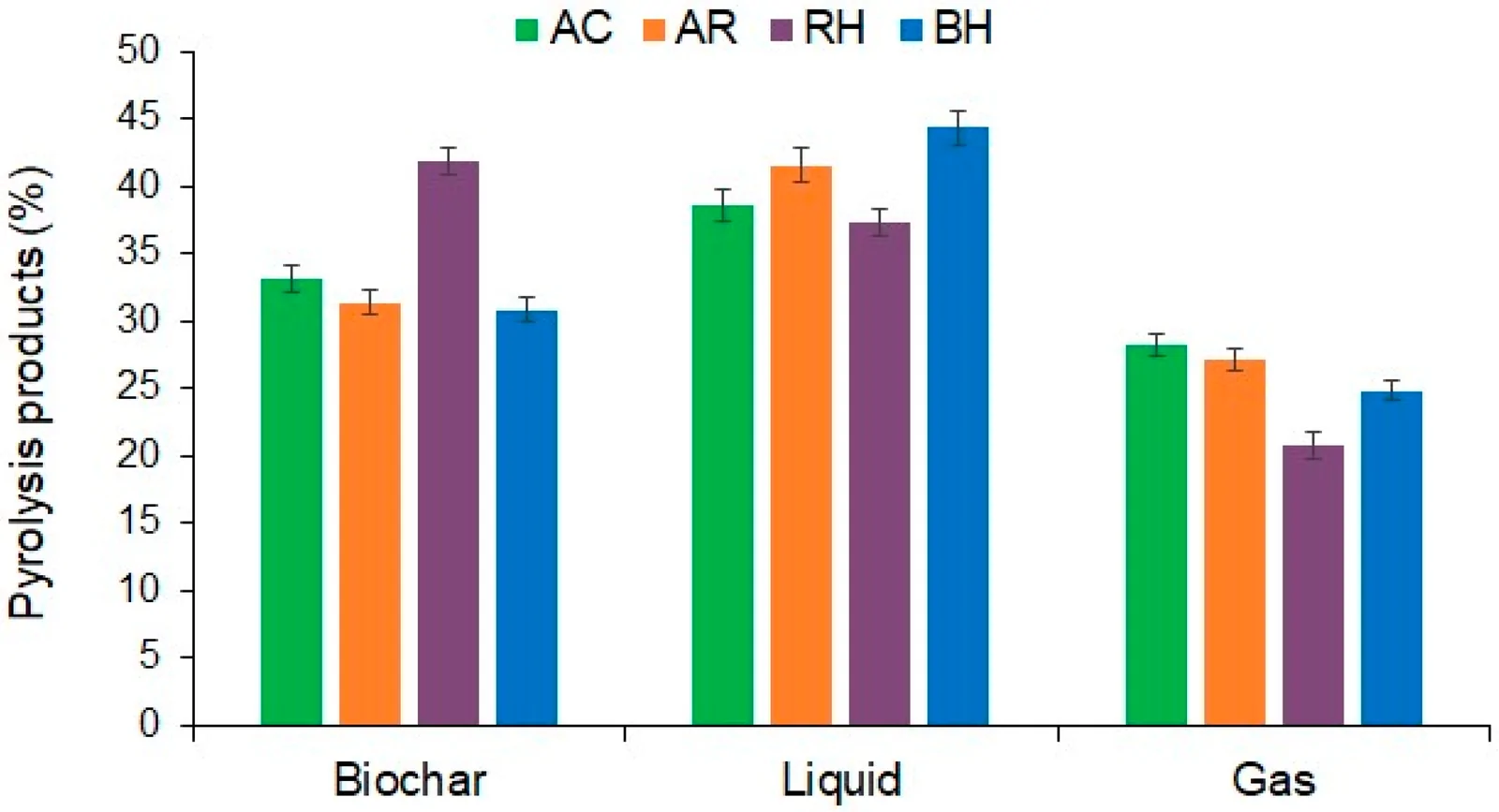
The composition of pyrolysis products (mean of three replicates; error bars represent ±3%).

Pyrolysis of biomass at 550 °C results in the formation of a solid residue (biochar) and pyrolysis gases, which are a mixture of condensable vapors (pyrolysis liquid) and non-condensable gases. The composition of the pyrolysis products was calculated according to [27] and is shown in Figure 1. Most of the pyrolysis products of the biomass samples are pyrolysis liquid, except for the RH sample, which contains more biochar. The yield of liquid, solid, and gaseous products is 37–44 wt%, 30–42 wt%, and 20–28 wt%, respectively.

According to studies, the pyrolysis temperature of 450–550 °C promotes the formation of liquid products to a greater extent [28]. The results obtained in this study were compared with other experimental data on the pyrolysis of plant biomass. The yield of pyrolysis products of AC is comparable to switchgrass and *Cenostigma pluviosum* [29,30], AR is comparable to *Alternanthera philoxeroides* [31], RH is comparable to Handroanthus sp. and banana pseudo-stem, during the pyrolysis of which a high mass yield of biochar is observed [30,32], and BH is comparable to sunflower husk pellet and *Acacia holosericea* trunk, which showed a high yield of pyrolysis liquid [33,34]. The difference in values can be explained by the composition of the feedstock and the influence of the pyrolysis process parameters.

### 2.2. Physicochemical Properties of Biochar

Adsorption indices can be determined by both the surface properties of the sorbents and the structural and chemical properties of the macromolecules, which influence chemisorption. The pyrolysis process significantly increased the content of fixed carbon in the feedstock and converted it into stable aromatic compounds. The main characteristics of the biochars obtained from the studied biomass are presented in Table 1. Thus, four samples were examined:

- *Amaranthus cruentus* biochar (ACB);
- *Amaranthus retroflexus* biochar (ARB);
- rice husk biochar (RHB);
- buckwheat husk biochar (BHB).

The highest amount of fixed carbon (FC) was found in the BHB sample, and the lowest in ARB. The ash content in the studied biochar samples varied from 7.19 to 43.53%. Mineral components can both improve and worsen the adsorption of mycotoxins. The BHB sample was characterized by the minimum ash content, while RHB had the maximum. The volatile matter (VM) content was ≈20%, with the exception of the RHB sample. The main chemical components of ACB, ARB, and BHB are potassium and calcium; their total content was 52.80–85.53% of the detected elements. The main elements of RHB are silicon and potassium with a content of 25.95% and 2.59%. Rice is known to absorb more silicon from the soil than any other plant [35], which explains its high content in RHB. Minor elements included magnesium, phosphorus, chlorine, sulfur, iron, manganese, zinc, bromine, and strontium. Because XRF data are semi-quantitative and do not include light elements (C, O, Na), they are used here only for a qualitative comparison of the samples.

**Figure 2:**
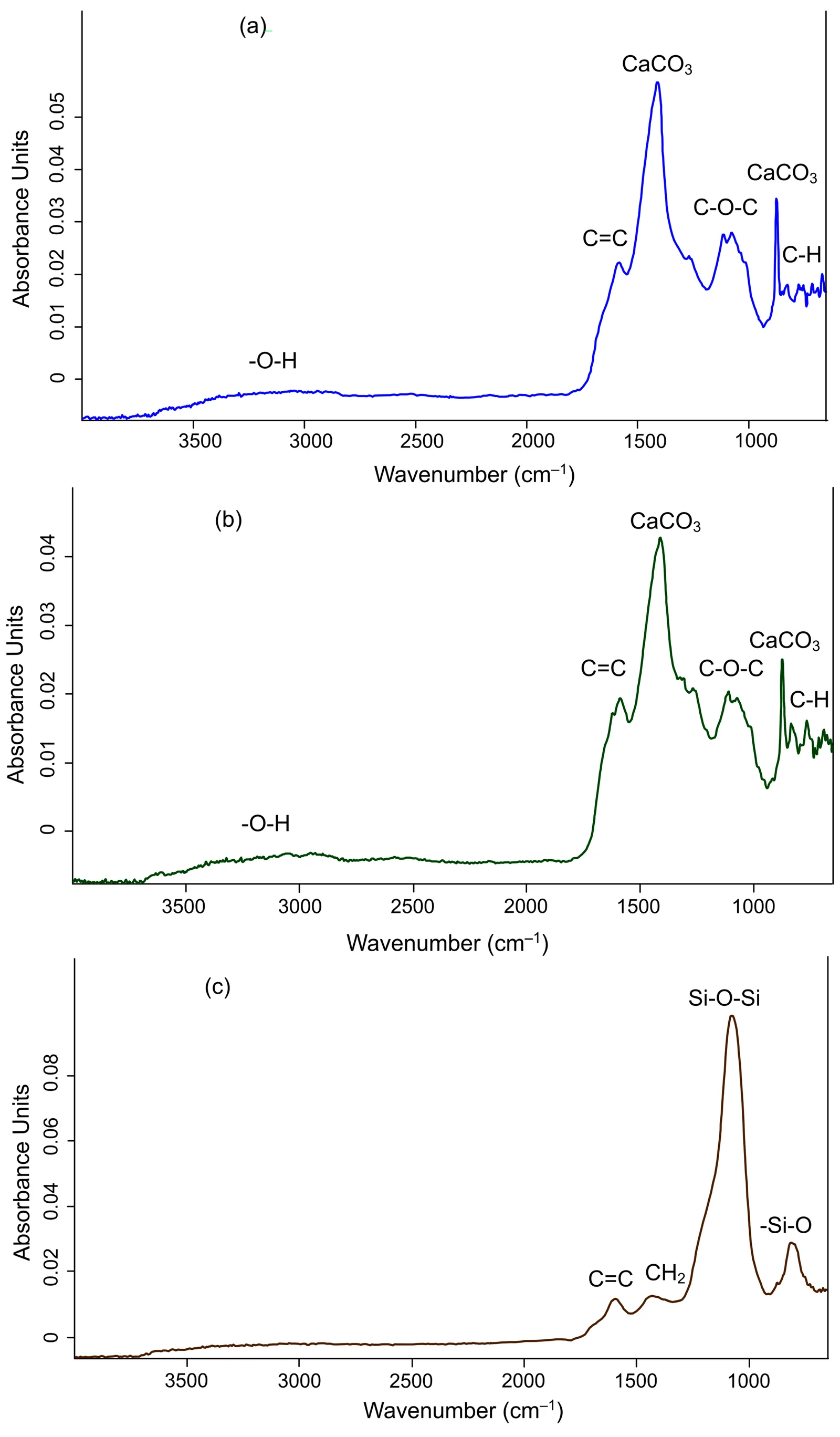

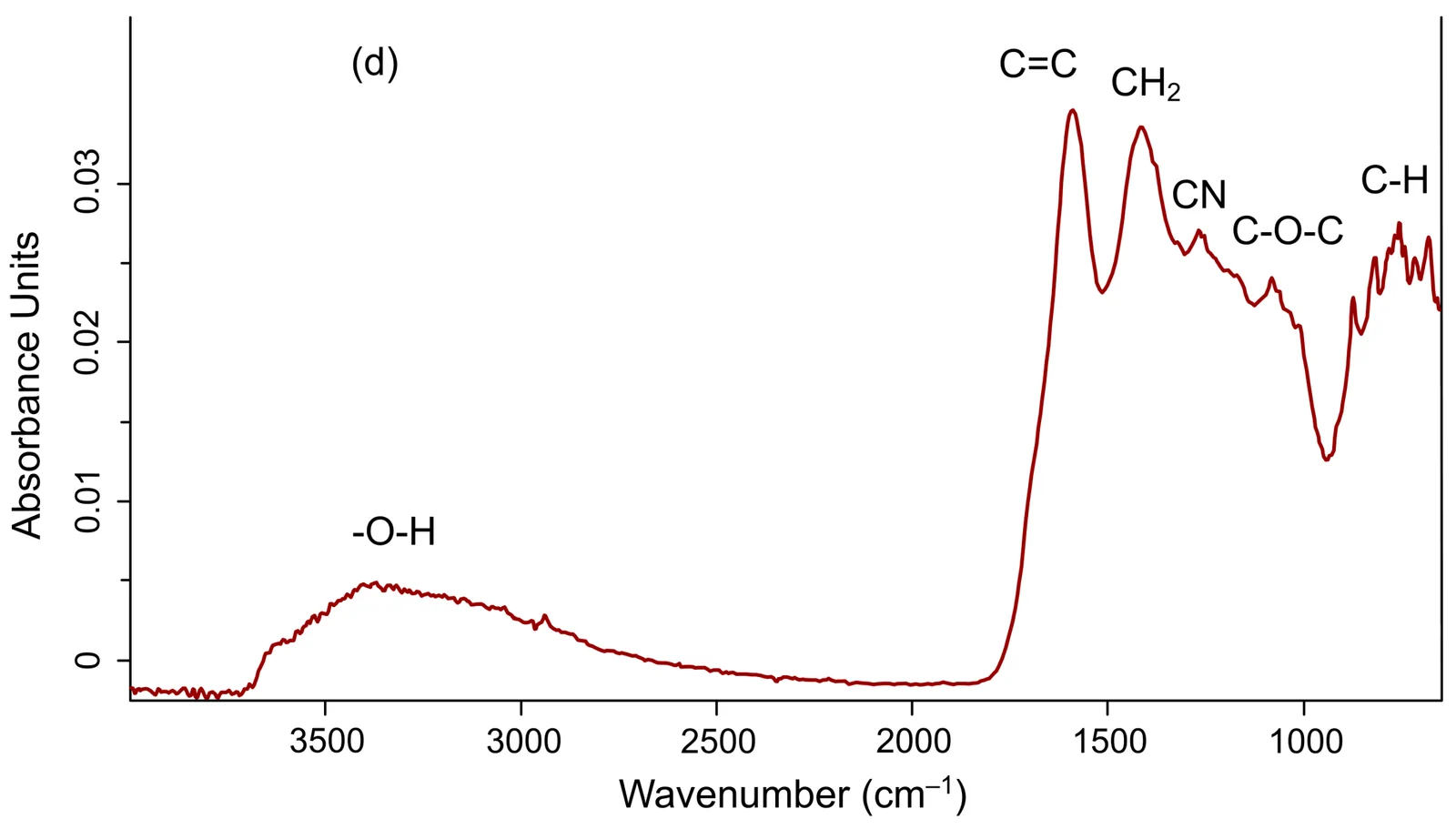
FTIR analysis of biochar samples: (**a**) ACB; (**b**) ARB; (**c**) RHB; (**d**) BHB.

FTIR analysis was performed to obtain an idea of the functional groups on the surface of biochars, since the adsorption properties of the samples vary depending on the presence of reactive functional groups. The results of the analysis of the samples are presented in Figure 2. FTIR spectra revealed the presence of various chemical functional groups in the biochars. Weak stretching vibrations of O–H bonds in alcohols, phenols, and residual water are observed in the range of 3000–3600 cm^−1^. These vibrations are most noticeable in the BHB sample. This indicates the preservation of phenolic or alcohol functional groups, as well as the possible presence of adsorbed water on the surface of the carbonized material. The bands at 1587–1594 cm^−1^ are associated with the stretching vibrations of C=C in aromatic rings, which indicates the presence of an aromatic group of lignin [36]. A clear peak of these vibrations is visible in the BHB sample. The bands at 1414–1437 cm^−1^ in BHB and RHB are assigned to CH_2_ bending vibrations [19]. During pyrolysis, some of the aromatic and aliphatic structures included in the carbon structure of the material are preserved. The bands in the range of 1410–1411 cm^−1^ and 874 cm^−1^ correspond to the spectra of calcite [37] in the ACB and ARB samples. The stretching vibrations of the C–N bond are observed at 1268 cm^−1^ in the BHB sample [38]. The bands at 1050–1150 cm^−1^ are associated with C–O–C stretching, clearly visible in ACB and ARB; in these P- and Si-containing samples, P–O and Si–O vibrations may also contribute. In the RHB sample, a significant vibrational peak is seen at 1100 cm^−1^, which corresponds to the antisymmetric stretching of Si–O–Si [38]. The vibrations of Si–O of silica are observed at 815 cm^−1^ for the RHB sample [19]. The peaks at 754–772 cm^−1^ in ACB, ARB, and BHB are assigned to out-of-plane C–H bending of aromatic rings [36]. At pyrolysis temperatures, most oxygen-containing groups are likely removed through dehydration and decarboxylation processes. This makes the biochar carbon more stable [39,40].

**Figure 3:**
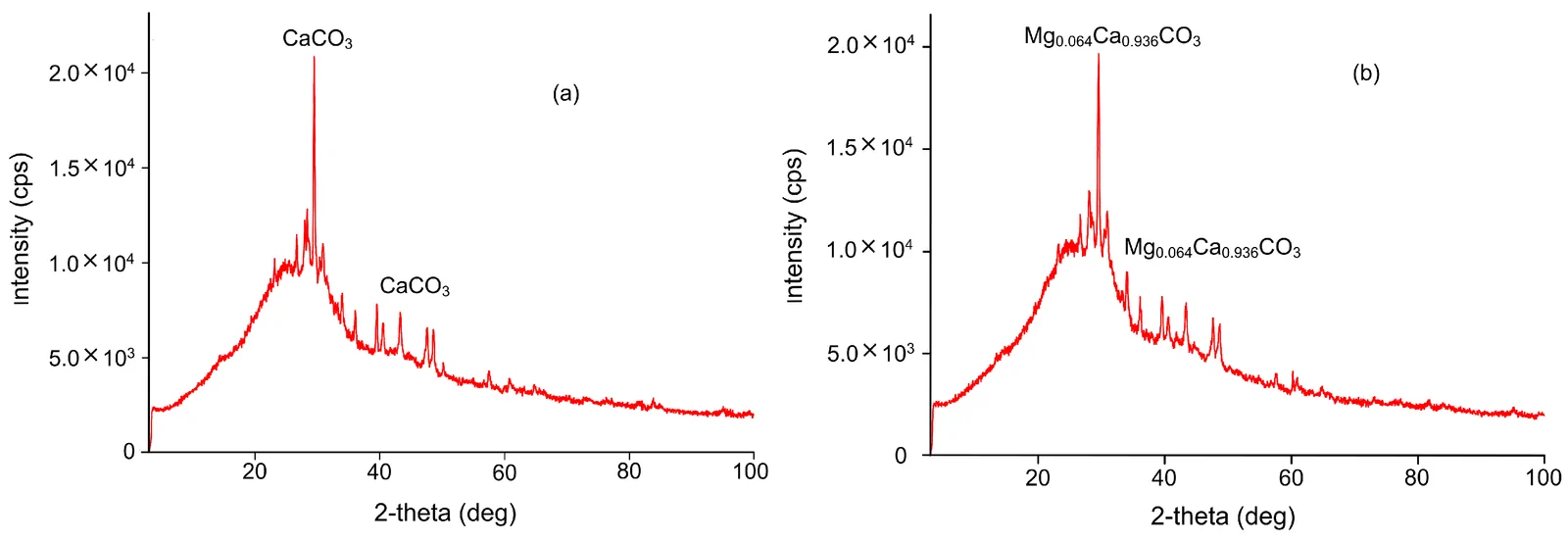

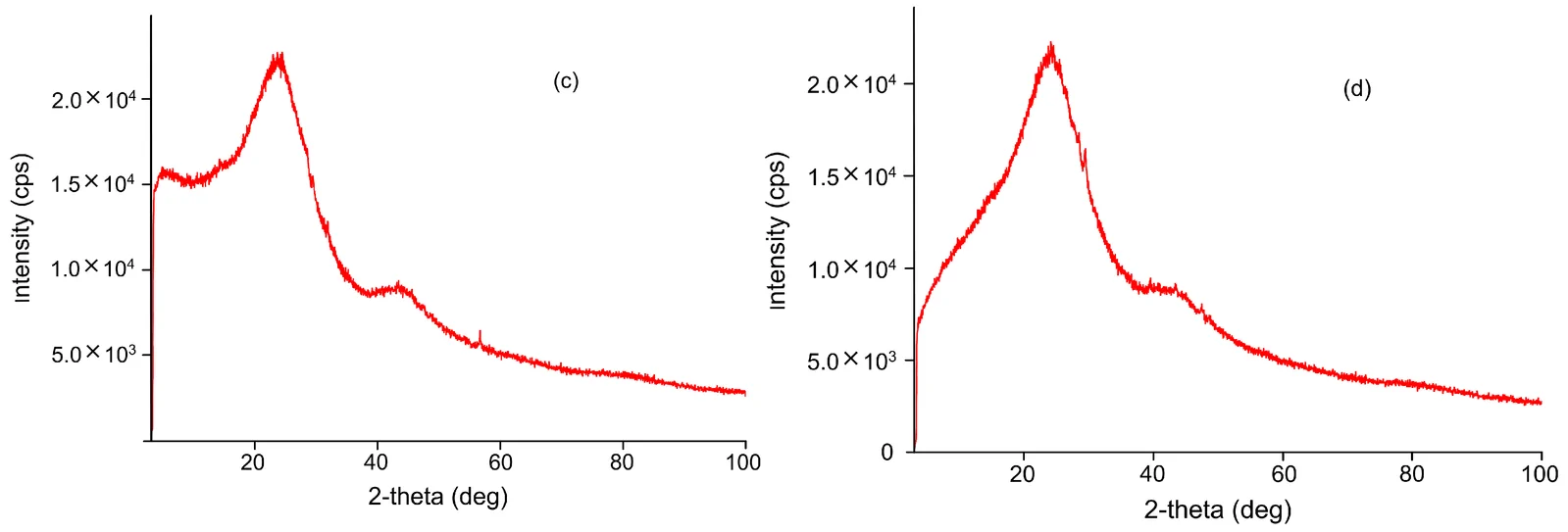
X-ray diffraction of biochar samples: (**a**) ACB; (**b**) ARB; (**c**) RHB; (**d**) BHB.

To evaluate the changes in the crystallinity of the biochar samples, X-ray diffraction analysis was used; the results are presented in Figure 3a–d. The main crystalline phase in the ACB sample is calcite (CaCO_3_) with a hexagonal structure (space group R-3c). The main peaks are at 2θ ≈ 29.5°, 39.5°, 43.2°, 47.6°. Calcium in the above-ground parts of amaranth is present mainly in the form of oxalate, which decomposes to carbonate during pyrolysis (CaC_2_O_4_ → CaCO_3_ + CO). Calcium carbonate in biochar is a typical ash component. [41,42]. The presence of peaks in the region of 2θ ≈ 25° indicates the occurrence of crystallization processes in materials that have not yet achieved a fully developed crystalline organization, but have increased structural order [43]. The presence of a peak at 2θ ≈ 43° indicates the formation of more complex and disordered carbon structures [44]. Thus, the ACB sample is characterized by a mixed lattice type with the presence of crystalline and amorphous phases. The X-ray amorphous part of the sample is probably disordered carbon (pyrolysis coke) formed during the decomposition of organic matter. Quartz, feldspars, and other soil-derived minerals were not detected, indicating very little soil contamination of the feedstock. No crystalline potassium phases were identified despite the high K content; potassium may be present in a poorly crystalline or finely dispersed form, or at concentrations below the detection limit.

The main crystalline phase of the ARB sample is magnesian calcite (Mg_0.064_Ca_0.936_CO_3_) with a hexagonal structure (sp. group R-3c). The main peaks are at 2θ ≈ 29.6°, 39.6°, 43.3°, 47.7°. Here, one can also assume that crystallization processes occur against the background of an incompletely developed crystalline organization, but an increased structural order in the material. The average size of calcite crystallites is 30–40 nm, which indicates good crystallinity while maintaining nanoscale dimensions. An amorphous carbon matrix (turbostratic carbon) is present and makes up approximately 50–70% of the total volume. During the flowering period, along with the concentration of calcium in the above-ground parts of amaranth, the plant may also contain an increased amount of magnesium [41].

The X-ray diffraction patterns of the biochar samples from buckwheat and rice husks were similar. The diffraction patterns had a diffuse baseline. Broad peaks at 2θ ≈ 20–30°, 41–46° indicate a progressive loss of cellulose crystallinity and the formation of more complex and disordered carbon structures [44]. The BHB sample was completely X-ray amorphous, i.e., the material was an amorphous carbon residue without significant mineral inclusions. The RHB sample was also X-ray amorphous, which is consistent with [45]. RHB was a two-phase amorphous system consisting of amorphous silica (SiO_2_) (the main mineral component) and amorphous turbostratic carbon. At pyrolysis temperatures <700 °C, silica retains an amorphous, “biogenic” structure. The simultaneously occurring carbonization of the organic matter of the original husk leads to the formation of an amorphous carbon residue (pyrolysis coke) with a turbostratic arrangement of carbon layers [35].

**Figure 4:**
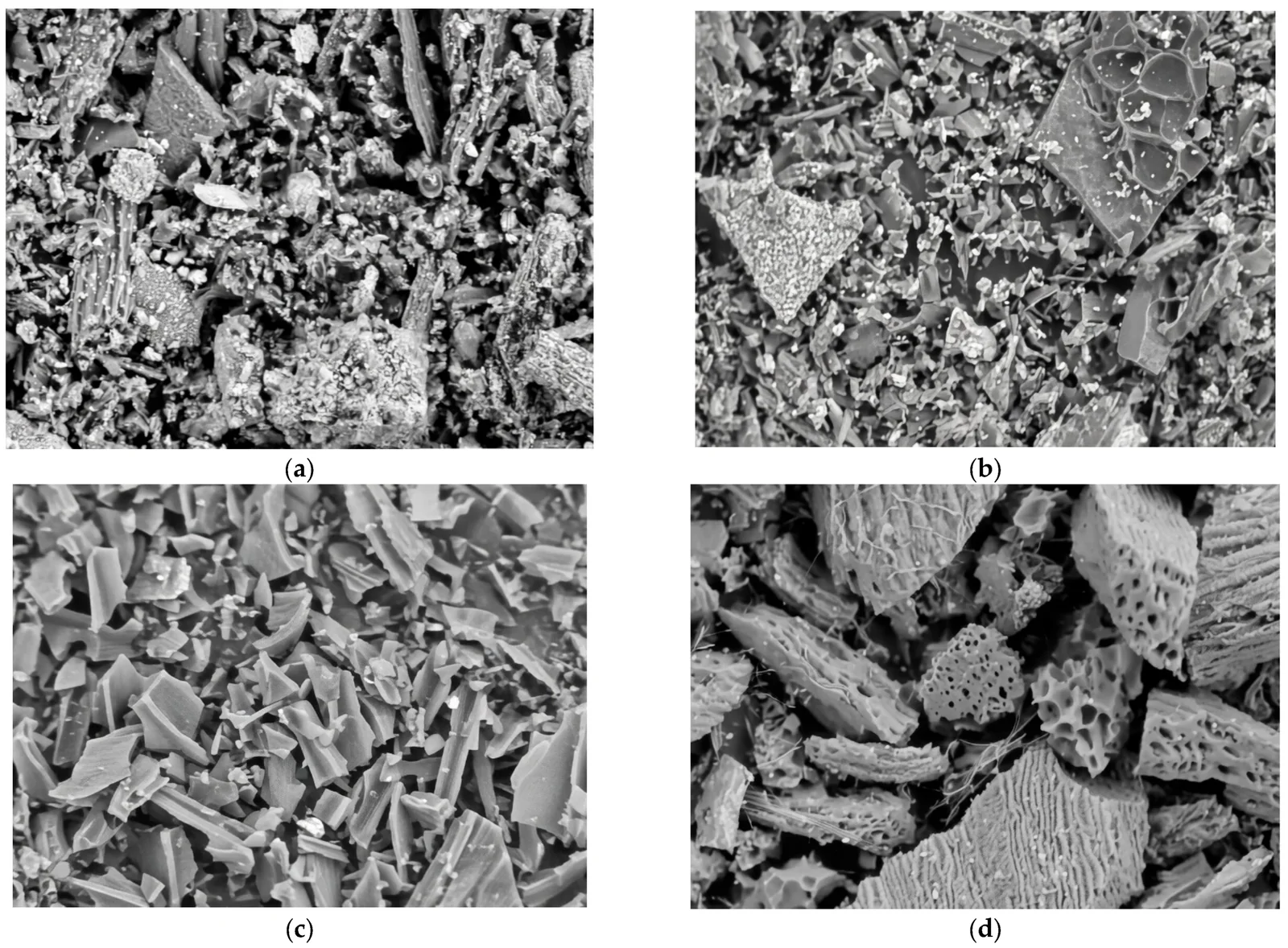
Morphological analysis of (**a**) ACB; (**b**) ARB; (**c**) RHB and (**d**) BHB.

Studying the surface morphology and pore structure of biochars is particularly important for predicting the effectiveness of their practical use. Figure 4 shows micrographs of pyrolysis biochar samples. During preliminary grinding, the fibrous structure of the original biomass was significantly destroyed. Heat also caused physical changes in the structure and cellular tissue of the biomass. Pyrolysis at 550 °C removed free and chemically bound water, and thermally decomposed the organic components of the biomass. Fine particles underwent partial sintering and amorphization.

The ACB and ARB samples retained some fibrous structure, with cracks and irregularities visible on the surface. The samples appear brittle and fractal due to the cracks. The ARB sample also exhibits some cellular structure. The RHB particles are aggregates consisting of smooth, layered structures. The BHB particles have a rough fibrous structure with distinct oval macropores formed by the removal of volatiles and moisture from the interior of the cells.

### 2.3. BET Surface Area and Pore-Size Distribution

**Figure 5:**
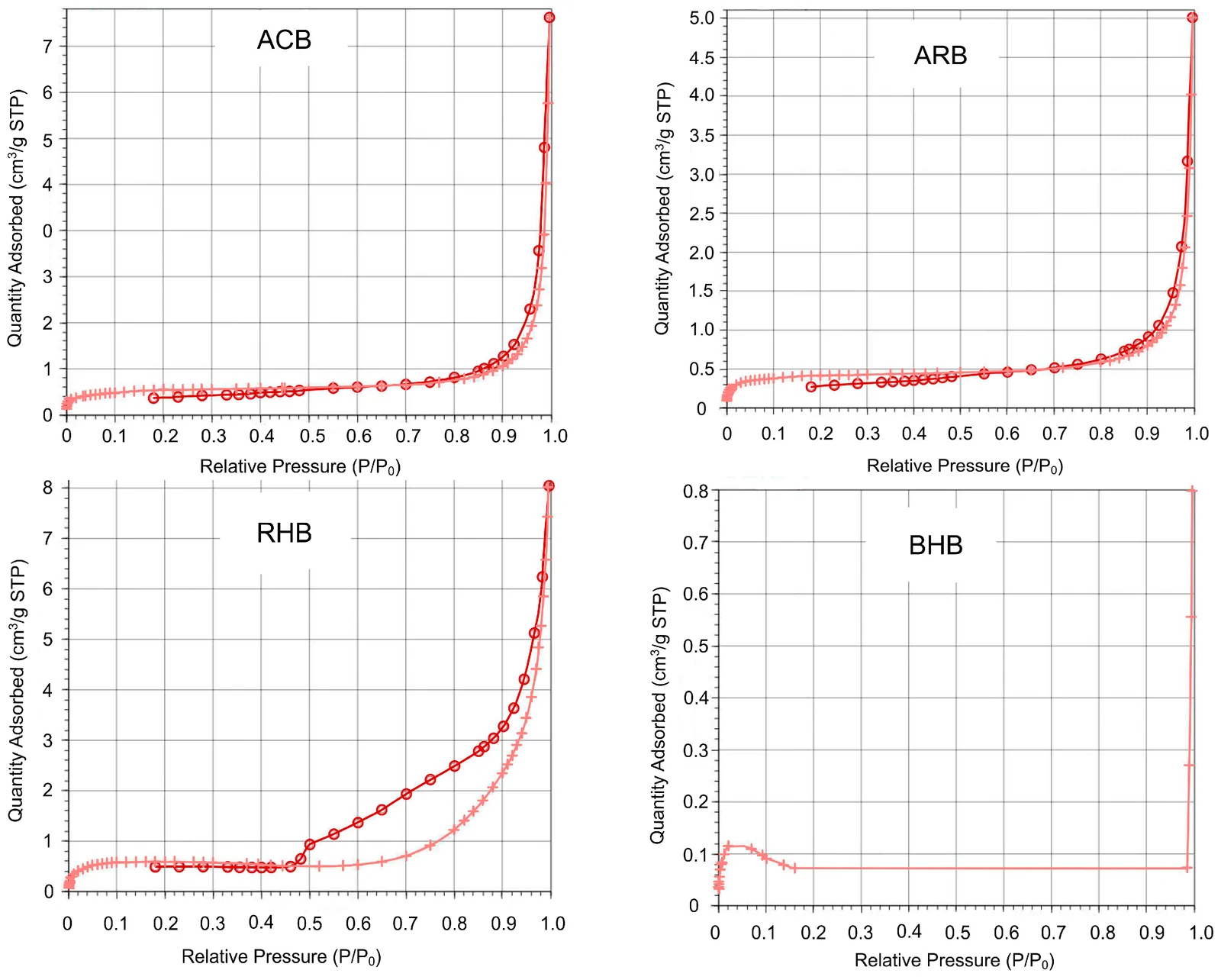
Nitrogen adsorption–desorption isotherms of the biochars. 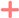
—adsorption, 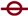
—desorption branches.

The specific surface area of the biochars was determined by low-temperature nitrogen adsorption–desorption (Figure 5, Table 2). According to the IUPAC classification [46], the isotherms of ACB and ARB were of Type II and showed low, nearly constant uptake (about 0.4–0.6 cm^3^ STP/g) over P/P_0_ = 0.05–0.7, followed by a sharp rise at P/P_0_ > 0.9 (to about 7.6 and 5.0 cm^3^/g at P/P_0_ ≈ 1, respectively). Such a profile is typical of non-porous or macroporous solids, on whose external surface multilayer adsorption occurs. Both samples exhibited only narrow hysteresis loops. The RHB isotherm was also of Type II but showed a pronounced H3 hysteresis loop that began at P/P_0_ ≈ 0.45 and persisted up to saturation; this indicates slit-shaped mesopores between plate-like particles or aggregates, consistent with the plate-like morphology revealed by SEM. Loop closure at this relative pressure is usually attributed to the tensile strength effect of liquid nitrogen, so pore-size data below ~4 nm should be interpreted with caution. The BHB isotherm was nearly flat (≤0.12 cm^3^/g up to P/P_0_ = 0.98), with a sharp rise only at P/P_0_ ≈ 0.99 and no distinct desorption branch, indicating that this sample is non-porous within the sensitivity of the method. However, SEM revealed a distinct honeycomb-like macroporous structure. This may indicate that the low nitrogen-accessible surface area reflects the length scale probed by this method rather than a complete absence of porosity in the sample.

The BET specific surface area was very low for all samples: 0.446 m^2^/g (BHB) < 1.527 m^2^/g (ARB) < 1.976 m^2^/g (ACB) < 2.295 m^2^/g (RHB) (Table 2). The t-plot analysis revealed only a minor micropore contribution: 0.16, 0.38, and 0.21 m^2^/g for ACB, ARB, and RHB, respectively (8.2, 24.9, and 9.1% of the BET surface area), whereas no micropores were detected in BHB. The Horvath–Kawazoe pore-size distributions of ACB, ARB, and RHB were dominated by the narrowest pores detected (0.66 nm), and the incremental micropore volumes did not exceed 2.1 × 10^−4^ cm^3^/g per size interval. Given the extremely small micropore surface area, these distributions are only indicative. Thus, the N_2_-accessible porosity of the biochars is formed almost entirely by meso- and macropores.

The S_BET_ values obtained (0.45–2.30 m^2^/g) are lower than those of similar plant-derived biochars produced at 400–550 °C. For example, values of 210.9 m^2^/g [47], 262.0 m^2^/g have been reported for rice husk biochars produced at 500 °C, 59.91 m^2^/g for rice straw biochar and 58.60 m^2^/g for apple branch biochar [25], 92.0 m^2^/g for wheat straw biochar produced at 550 °C [26], 17.02 m^2^/g for buckwheat husk biochar produced at 550 °C [48], and 11.96 m^2^/g for corn stalk biochar produced at 400 °C [49]. It should be noted that the demineralization of wheat straw biochar (ash content reduced from 28.1 to 4.3%) increased its S_BET_ 2.7-fold [26]. Note that the measured S_BET_ of biochars depends strongly on the degassing temperature [50]; therefore, the comparison with the literature is semi-quantitative.

**Figure 6:**
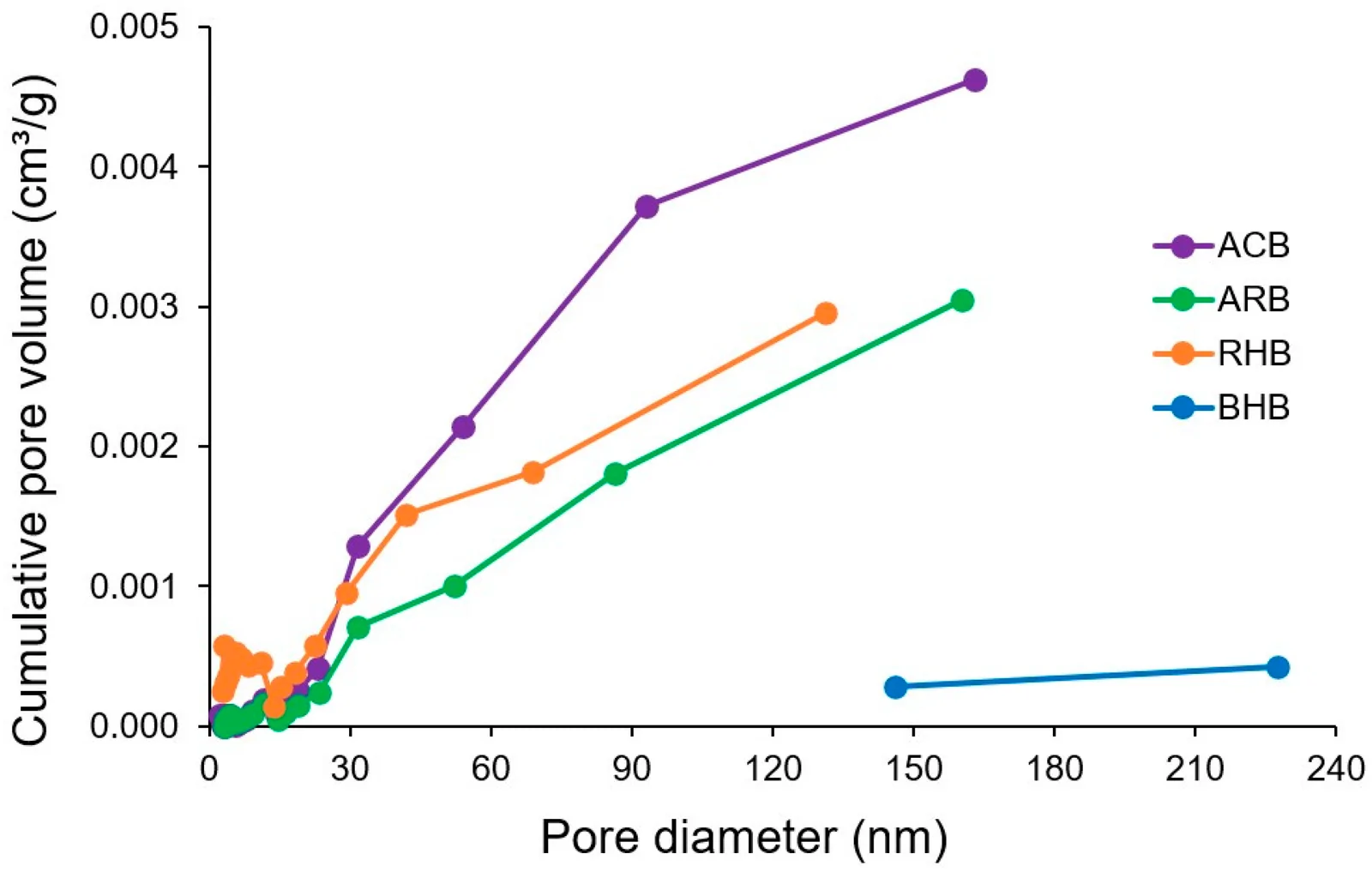
Cumulative BJH pore volume of the biochars as a function of pore diameter (desorption branch; for BHB, the adsorption branch is shown because no desorption branch was recorded).

The meso- and macropore size distributions calculated by the BJH method (Figure 6) also differed between samples. In ACB and ARB, macropores larger than 50 nm accounted for 75–79% of the total pore volume (0.0134 and 0.0078 cm^3^/g), whereas pores of 3–11 nm accounted for only 4–7%. In contrast, RHB had a broader distribution with a substantial mesopore fraction (62% of the pore volume in the 2–50 nm range; about 31% in the 3–11 nm range) and a comparable total volume (0.0125 cm^3^/g). In BHB, pores were detected in only two size classes (146–228 nm), with a total volume of 0.0007 cm^3^/g. It should be noted that S_BET_ values below 5 m^2^/g approach the practical accuracy limit of volumetric nitrogen adsorption at 77 K, and the cumulative BJH surface area of RHB (about 4 m^2^/g) exceeded the BET value; this is probably an artifact associated with the desorption branch below 4 nm. Therefore, these data should be regarded as semi-quantitative, and the BHB surface area as an estimate at the sensitivity limit of the method.

It is important to note that specific surface area is not an unambiguous indicator of adsorption capacity, since sorption is also governed by a combination of factors, including solution chemistry, pore-size distribution, and the presence of surface functional groups [49].

### 2.4. Iodine Adsorption Properties of Biochar

In this study, we compared the adsorption performance of the biochar using I_2_/I_3_ because the adsorption amounts of iodine are widely used as indicators to assess the adsorption capacities of biochar [51]. The results are presented in Table 3.

The highest iodine uptake (291 and 335 mg/g) was recorded for the amaranth biochars, whereas the values for RHB and BHB were two to three times lower. The similar values for ACB and ARB are consistent with the similar composition of their feedstocks. Surface area and pore structure are known to affect iodine adsorption [53]; iodine is adsorbed predominantly in micropores [54], and the iodine number correlates with the micropore characteristics of carbon materials [55]. However, the values obtained in this work (108–335 mg/g) are inconsistent with the extremely low N_2_-accessible surface area (0.45–2.30 m^2^/g). Consequently, iodine uptake by these biochars cannot be unambiguously regarded as an indicator of microporosity. The measured iodine number can be affected by adsorbed volatiles, sulfur, and water-extractable components [56]. Chemical interaction of iodine with the alkaline Ca- and K-rich ash, as well as with reducing surface groups of the char, may contribute to this process [57]. This is supported by the fact that the highest values were obtained for the alkaline Ca/K-rich amaranth biochars and the lowest for the low-ash BHB.

### 2.5. Analysis of Zearalenone Adsorption by Biochars

In most cases, the removal of mycotoxins from aqueous media by adsorption is highly dependent on the pH of the medium, which influences binding mechanisms and structural changes. It is important that mycotoxins are not released from the adsorbent when the pH changes in different parts of the gastrointestinal tract (e.g., from the acidic environment of the stomach to the alkaline environment of the intestine). Under the acidic conditions of the first stage (nominal pH 2.0), ZEN was effectively adsorbed by three of the four biochars. The adsorption efficiency (*A*), desorption (*D*), and desorption-corrected adsorption efficiency (*A_F_*), together with the corresponding uptake at the tested dose (*q*, mg ZEN per g biochar), are presented in Table 4 (mean values; differences between samples were statistically significant at *p* ≤ 0.05).

The uptake q was calculated from the known mass of ZEN added to the sample (0.05 mg in 10 mL, i.e., an initial concentration of 5 µg/mL; toxin-to-sorbent mass ratio of 1:1000) and the sorbent mass (50 mg): *q* = *A*(%)/100 × (0.05 mg/0.05 g) = *A*(%) × 0.01 mg/g; *q*(*A_F_*) was calculated in the same way from *A_F_*(%). In this section, “adsorption efficiency” refers to the percentage of ZEN removed from solution under the stated conditions (at a fixed initial concentration of 5 µg/mL), and *q* is the uptake at this particular loading; it is dose-limited (maximum 1 mg/g) and is not an equilibrium adsorption capacity.

As shown in Table 4, ACB, ARB, and RHB adsorbed ZEN in vitro at 37 °C, with adsorption efficiencies of 90.5%, 77.3%, and 65.8% (corresponding to *q* = 0.905, 0.773, and 0.658 mg/g). Desorption did not exceed 1.5% of the bound toxin, indicating strong binding. BHB showed very low adsorption efficiency (10.6%, *q* = 0.106 mg/g) and was ineffective under the test conditions.

The desorption-corrected efficiency (*A_F_*) is important for evaluating an adsorbent, because it reflects the fraction of mycotoxin that remains bound when conditions change along the gastrointestinal tract, as opposed to transient binding followed by release. ZEN binding remained stable after the medium was shifted to pH 8.0.

Note that the pH of the suspensions after sorbent addition was not measured. Because biochars rich in calcium and potassium carbonates (ACB and ARB) can partially neutralize an acidic medium, the actual pH during the adsorption stage may have exceeded the nominal value of 2.0. The results should therefore be interpreted bearing in mind that the biochar itself may alter the pH; possible transformation of ZEN at elevated pH was not assessed.

It is of interest to compare the adsorption efficiency of the studied samples with that of mineral and organic enterosorbents such as montmorillonite, modified aluminosilicates, shungite, lignin, and zeolite, as well as commercial products such as Novasil Plus, Escent^®^ S, and Mycosorb^®^ (Table 4). Direct comparison with literature data requires caution, because the cited studies differ substantially in ZEN concentration, sorbent-to-toxin ratio, pH and duration of the digestion stages, incubation temperature, and analytical method (HPLC with fluorescence detection, LC-MS/MS, or ELISA). There is no single threshold in the literature that defines “significant” or “effective” in vitro adsorption: binding-efficiency thresholds range from >50% (for simultaneous binding of several mycotoxins) [62] to >85–90% (for high-surface-area sorbents designed to bind a single toxin) [19], depending on the toxin, matrix, and protocol. Therefore, the apparent advantage of ACB, ARB, and RHB over a number of commercial and modified sorbents should be regarded only as an indication of their overall competitiveness.

### 2.6. General Toxicity of Biochar

The ciliates can be used as a toxicity indicator [65]. In this study, ciliates were used to preliminarily assess the potential toxic impact of biochar on the protozoan community. Recent studies have increasingly highlighted the potential of ciliates to enhance monitoring precision due to their diverse ecological niches, rapid reproduction rates, and sensitivity to a wide range of pollutants [66]. When determining overall toxicity, the following test responses were taken into account: changes in the number of ciliates (survival). The results are presented in Table 5. Samples were classified as “toxic” at survival below 40%, “slightly toxic” at 40–69%, and “non-toxic” at 70% or higher. Values are means of five replicates (microaquariums); the ± values denote the measurement uncertainty of the method (Section 4.7).

As can be seen, the survival rate of ciliates was over 70% in both the aqueous acetone extract and the water extract. The data obtained are comparable to the general toxicity of a food product—oat flakes [67]. The highest survival rate of ciliates was observed with ACB biochar.

The *Stylonychia mytilus* survival test is a preliminary screening method for acute toxicity that allows potentially hazardous samples to be identified rapidly; however, on its own it cannot serve as comprehensive evidence that biochar is safe as a feed additive. As shown in a number of studies, ciliate bioassays are highly sensitive to a wide range of pollutants and can be used as the first tier of a multi-tier safety assessment [65,66]. Future studies should therefore assess potentially toxic metals, PAHs and other leachable contaminants, and include other relevant biological/animal test systems.

### 2.7. Relationship Between Physicochemical Properties of Biochar and ZEN Adsorption

**Figure 7:**
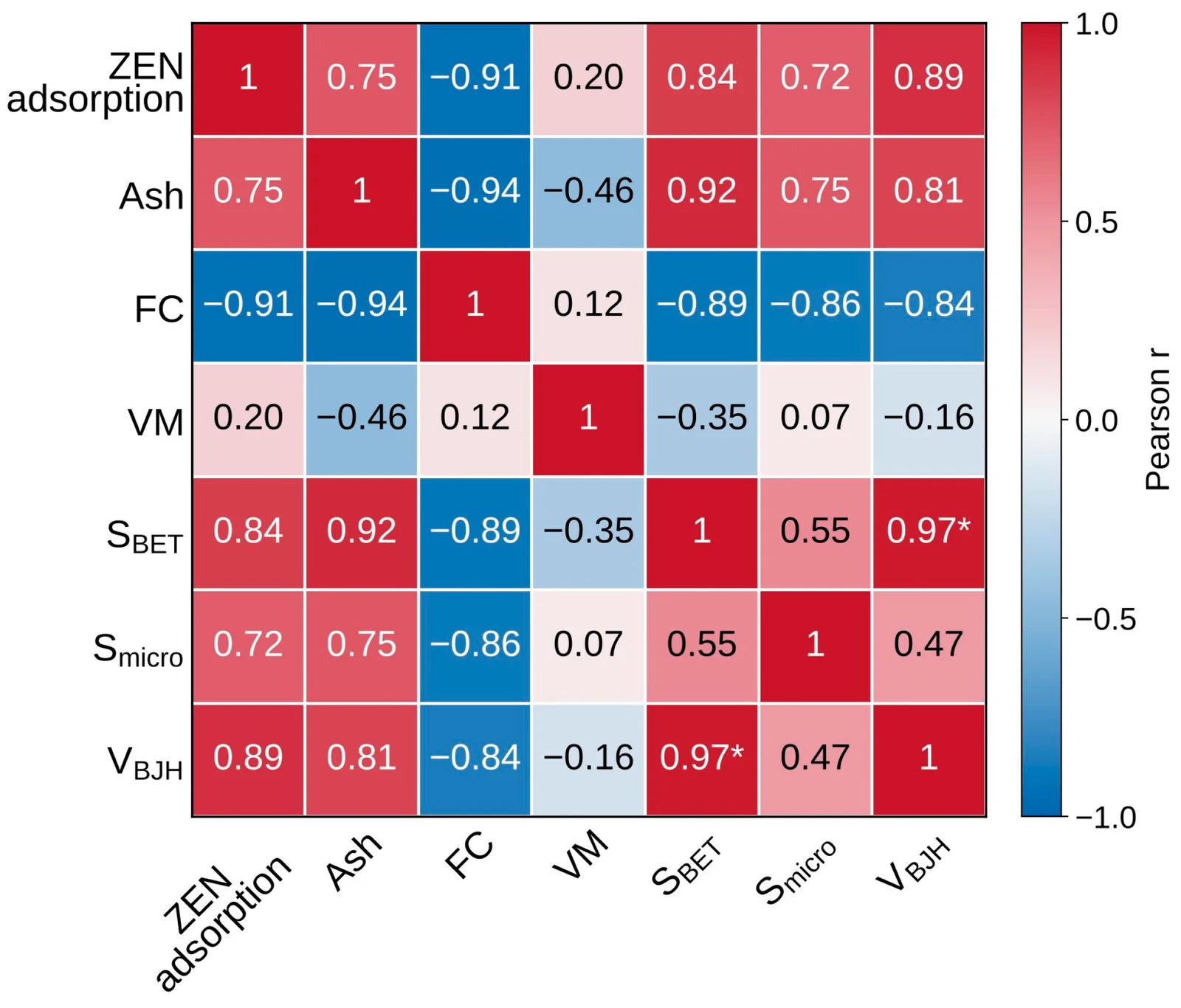
Pearson correlation matrix of ZEN adsorption efficiency, chemical composition, and textural characteristics of the four biochars (*n* = 4). * Coefficients significant at *p* < 0.05.

The ZEN adsorption efficiency of the biochars decreased in the order ACB > ARB > RHB > BHB (90.5% > 77.3% > 65.8% > 10.6%, respectively). To identify the properties underlying this order, adsorption efficiency was compared with the proximate composition and ash composition (Table 1) and with porosity (Table 2) using Pearson correlation (Table 6 and Table 7, Figure 7). Because the ZEN dose and sorbent mass were the same in all experiments, the coefficients for removal efficiency (*A*) and uptake (*q*) were identical. With only four samples, this analysis is descriptive.

For all four samples (*n* = 4), ZEN removal correlated negatively with fixed carbon (FC) content (*r* = −0.91; *p* = 0.086) and positively with BJH pore volume (V_BJH_) (*r* = 0.89), BET specific surface area (S_BET_) (*r* = 0.84), ash content (*r* = 0.75), and micropore area (S_micro_) (*r* = 0.72), whereas the correlation with volatile matter (VM) was weak (*r* = 0.20) (Table 7, Figure 7). None of these coefficients reached the critical value for four samples (|*r*| = 0.950 at *p* < 0.05). The coefficients are largely driven by a single sample, BHB, which differs from the other biochars in almost every parameter: it has the lowest ash content (7.2%), the highest FC content (72.1%), a surface area at the sensitivity limit of the method, and the lowest adsorption.

Excluding BHB reversed the sign of several correlations (Table 7). For ACB, ARB, and RHB, ZEN removal decreased with increasing ash content (*r* = −0.99) and increased with increasing volatile matter (*r* = 0.90). The correlation with S_BET_ became weak and negative (*r* = −0.38): RHB, which had the highest S_BET_ (2.30 m^2^/g), was less effective than ACB (1.98 m^2^/g) and ARB (1.53 m^2^/g). However, in this subgroup the highest ash content coincides with a change in ash type, from Ca/K carbonates (ACB and ARB) to Si-rich ash (RHB). Because statistical testing is not possible with *n* = 3, these results should be regarded only as indicative trends.

Qualitatively, the two most effective biochars (ACB and ARB) had a high ash content dominated by K and Ca, with calcite or magnesian calcite as the main crystalline phase. RHB, whose ash consisted mainly of amorphous silica, was less effective. BHB showed the lowest efficiency: although its ash was also dominated by K and Ca, ash accounted for only 7.2% of the material, and no crystalline mineral phase was detected. This pattern is consistent with a contribution of carbonate-containing ash to ZEN binding.

Among the properties of the biochars themselves, a statistically significant correlation (*p* < 0.05; *n* = 4) was found only between S_BET_ and V_BJH_ (*r* = 0.97). This indicates that the surface of these materials is formed by meso- and macropores. The strong negative correlation between ash content and fixed carbon (FC) (*r* = −0.94) arises because FC was calculated by difference, and this relationship did not reach statistical significance.

## 3. Discussion

Interest in low-cost carbon-containing adsorbents such as biochar is currently growing [20,40,68]. Crop waste is of particular interest for biochar production, as it has a “clean” composition and mostly does not contain toxic substances. Furthermore, crop waste accounts for 35.6% of the total amount of agricultural waste, i.e., it is second in volume after livestock waste [69].

Amaranths AC and AR are unique plants that belong to the C4 class, so they are capable of photosynthesis at high temperatures. Some species of amaranth are cosmopolitan plants, capable of growing in any climate zone, including the cryolithic zone [70]. Liquid and solid products of amaranth pyrolysis have shown unusual properties [71], so a decision was made to study them in detail and expand the scope of their application. Rice husk is a large-tonnage waste, the processing of which poses certain difficulties associated with the high abrasiveness of the particles, caused by their high silicon dioxide content, which makes up the bulk of the husk ash [72,73]. In the rice husk used in this study, the ash content was 18.52 wt%, and Si was the dominant element in the ash of RHB. We also previously conducted pyrolysis of buckwheat husks. The solid pyrolysis product showed low ash content of ≈7% and high carbon content of ≈81% [27]. This is a very interesting result, so biochar was also considered as a research subject.

Our previous studies demonstrated the feasibility of producing biochar from non-food plant materials; however, its use as an adsorbent for mycotoxins had not been previously considered. The efficiency of mycotoxin adsorption is determined by a combination of factors: the surface properties of the adsorbents and the structural and chemical properties of the macromolecules that influence chemisorption [74,75]. These include the properties of the adsorbent itself (surface properties, structural and chemical properties of the macromolecules, morphology, and porosity) and external conditions (temperature, pH, contact time) [76]. This study analyzed the physicochemical characteristics of biochars and their adsorption capacity for ZEN, as well as their overall toxicity.

In this work, the hypothesis that the mineral fraction plays the leading role was tested on three points. The first point concerned porosity. All biochars were practically non-porous to N_2_ (S_BET_ = 0.45–2.30 m^2^/g); their pore volume is formed by meso- and macropores, and the micropore contribution is negligible. Nevertheless, three samples (ACB, ARB, and RHB) removed ZEN effectively (65.8–90.5%). With *n* = 4, ZEN removal correlated positively but non-significantly with S_BET_ (*r* = 0.84); however, this relationship was driven by a single sample, BHB, which simultaneously had the lowest ash content, surface area, and adsorption efficiency. Among the high-ash biochars, the relationship was weak and negative (*r* = −0.38): RHB, with the highest S_BET_, was inferior to ACB and ARB. The amount of ZEN normalized to surface area was 0.74–1.59 µmol/m^2^, corresponding to approximately 45–96% of a monolayer on the N_2_-accessible surface (assuming about 1 nm^2^ per molecule). Such high coverage at concentrations of the order of µg/mL requires either a high surface affinity for ZEN or the involvement of binding sites not detected by N_2_ physisorption: ultramicropores inaccessible to N_2_ at 77 K (CO_2_ adsorption was not performed) or mineral surfaces exposed upon wetting [50]. Consequently, physical adsorption in N_2_-accessible pores alone does not explain the differences between the samples.

The second point concerned the degree of carbonization. FC content was not associated with high adsorption efficiency: the most carbonized sample, BHB (FC = 72.1%), was the least effective, and with *n* = 4 ZEN removal correlated negatively with FC (*r* = −0.91; *p* = 0.086). Because FC is calculated by difference, this relationship largely reflects the relationship with ash content.

The third point concerned ash type. The ash of ACB and ARB consisted mainly of K and Ca and contained calcite or magnesian calcite, whereas the ash of RHB was dominated by amorphous silica; BHB contained only 7.2% ash and had no crystalline mineral phases. The order ACB > ARB > RHB > BHB corresponds to a transition from carbonate Ca/K ash to siliceous ash and then to a low-ash material. When BHB was excluded, ZEN removal decreased with increasing ash content (*r* = −0.99); however, this does not contradict the hypothesis, because in this subgroup the highest ash content (RHB) coincides with a change in ash type. This result may indicate that what matters is not the ash content as such but the nature of the mineral phase. The appreciable efficiency of RHB (65.8%) despite its low Ca and K content may be related to its larger mesopore fraction (about 31% of the pore volume in the 3–11 nm range versus 4–7% for the amaranth biochars) and/or to the siliceous mineral phase, which requires separate investigation.

Thus, all three points of the hypothesis were satisfied at a qualitative level, but none of the correlations reached statistical significance. The hypothesis can be considered tentatively supported but not proven: with four samples, the biochar properties co-vary (ash content, FC, and S_BET_ are interrelated), and their contributions cannot be separated statistically.

ACB and ARB ranked differently in the two tests: ACB removed more ZEN (90.5 vs. 77.3%), whereas ARB had a higher iodine number (335 vs. 291 mg/g). If iodine uptake by these biochars is governed largely by reactions with the alkaline ash rather than by porosity, the higher iodine number of ARB would be consistent with its higher ash content (34.1 vs. 27.7%). The reasons for the higher efficiency of ACB remain unclear; the accessibility of the carbonate surface and differences in the organic matrix may contribute.

An important limitation is that the pH of the suspensions during the adsorption stage was not controlled. The carbonate-rich amaranth biochars may have partially neutralized the acidic medium; therefore, the association between carbonate Ca/K ash and ZEN removal may partly reflect a pH effect.

From a practical standpoint, all biochars were non-toxic in the *Stylonychia mytilus* test, and the in vitro efficiency of ACB is comparable to that of a number of commercial sorbents, although direct comparison is limited by differences in protocols.

Future studies should include ZEN adsorption isotherms and kinetics on ACB, which would allow the contributions of ion exchange, electrostatic attraction, surface precipitation, and complexation to be assessed; for many biochars, isotherms are better described by the Freundlich model, reflecting the heterogeneity of the surface formed during pyrolysis [77]. The most direct test of the hypothesis would be experiments with demineralized biochar and with pure mineral phases (calcite, amorphous silica) at controlled pH, with a complete ZEN mass balance. In addition, CO_2_ adsorption to assess ultramicroporosity, determination of potentially toxic elements and PAHs, evaluation of the potential for activation, and in vivo studies are needed.

## 4. Materials and Methods

### 4.1. Preparation and Characteristics of Raw Materials

**Figure 8:**
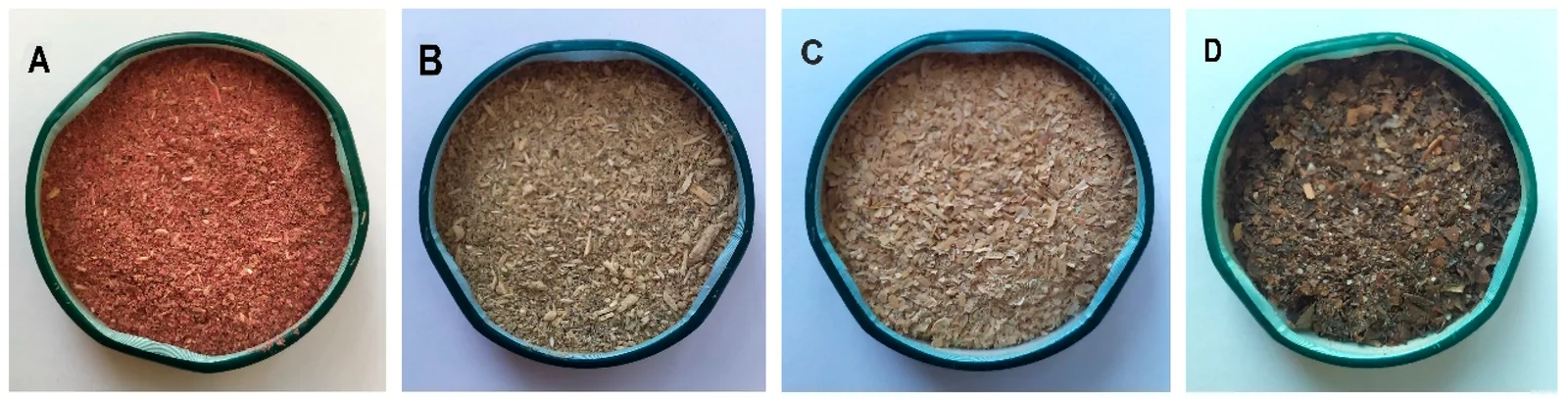
Photos of initial biomass samples: (**A**) AC; (**B**) AR; (**C**) RH; (**D**) BH.

Herbaceous biomass (AC and AR) was grown in a field with cultivated plants. They were then separated into leaves, inflorescences (some with seeds), and stems. Waste (RH and BH) was obtained after processing rice and buckwheat at a grain mill (Kazan, Russia). All samples were manually cut into small pieces with the help of blades and then finely powdered using a mixer-cum-grinder. Photos of the original biomass samples are shown in Figure 8.

Biomass samples were pre-dried at 105 °C in a drying chamber (ShSL-43/250 V, AnalytPromPribor, Moscow, Russia). The elemental composition was determined on an elemental analyzer (Euro EA 3000, Eurovector, SpA, Milan, Italy). Samples were weighed in tin capsules on a Sartorius CP2P microbalance (Sartorius AG, Göttingen, Germany). Callidus 4.1 software was used to evaluate the obtained data. Moisture, ash, and volatile matter content were determined in a muffle furnace (PMLS-2/1200, Milaform, Kazan, Russia) in accordance with [78,79,80,81]. The chemical compositions of biochars were studied using the EDX-800HS2 energy-dispersive fluorescence X-ray spectrometer manufactured by Shimadzu (Shimadzu, Kyoto, Japan) by a semi-quantitative method. Table 8 presents the physicochemical properties of the initial biomass samples.

### 4.2. Biochar Production Procedure

The process of obtaining pyrolysis biochar was carried out in a metal tubular reactor with a length of 28 cm and an internal diameter of 34 mm (Institute of Power Engineering and Advanced Technologies, Kazan, Russia) [82]. A 20 g sample of plant biomass (weighing accuracy ±0.0001 g) was placed in the reactor, which was then installed in the furnace. The outlet pipe of the reactor was connected to the condensation system. The temperature regime of the process included an initial temperature of 25 °C, heating to a final temperature of 550 °C at a rate of 10 °C/min. Temperature changes were monitored by a thermocouple connected to a data acquisition system, allowing for highly accurate recording of heating dynamics. All measurements were carried out in an atmosphere of inert nitrogen at a constant gas flow rate of 20 L/h. Pyrolysis products (volatile compounds) were fed into a condensation system. The liquid fraction was collected in a receiving flask, and non-condensable gases were diverted to a gas collection bag connected to the condenser outlet. After completion of the process and system cooling, the solid residue in the reactor was weighed. Each experiment lasted 2 h. All experiments were repeated three times, and the averaged mass balance data are presented in the article.

### 4.3. Analysis of the Biochar Physicochemical Properties

#### 4.3.1. IR Spectroscopic Analysis

IR spectra in the range (400–4000 cm^−1^) were obtained on a Bruker Vertex 70 FTIR spectrometer (Bruker Optik GmbH, Ettlingen, Germany), with the cuvette compartment purged with dry, purified air to remove water vapor. Interferograms were recorded with a resolution of 2 cm^−1^ and averaged over 128 scans. Spectra of solid samples were recorded using an ATR attachment with a ZnSe crystal (MIRacle, PIKE Technologies, Madison, WI, USA).

#### 4.3.2. X-Ray Diffraction Analysis

Powder X-ray diffraction analysis of biochar samples was performed using a MiniFlex 600 diffractometer (Rigaku, Tokyo, Japan) equipped with a high-speed D/teX detector. Measurements were performed using Cu Kα radiation (30 kV, 15 mA). The results were obtained at room temperature over a 2θ range of 3° to 100° with a step size of 0.02° and an exposure time of 0.24 s at each point without rotation.

#### 4.3.3. Scanning Electron Microscopy

Analysis of changes in the morphological structure of biochar samples was performed using a HITACHI TM-1000 SEM microscope (Hitachi High-Tech Corporation, Ibaraki, Japan) with a magnification of 10,000 and a resolution of 30 nm, an accelerating voltage of 15,000 V, and an emission current of 34.2 μA.

### 4.4. BET Surface Area and Pore-Size Distribution Analysis

The specific surface area and pore-size distribution of the biochars were determined by low-temperature nitrogen adsorption–desorption using an ASAP 2020 analyzer (Micromeritics Instrument Corp., Norcross, GA, USA) at the D.I. Mendeleev Shared Research Facility Center (D.I. Mendeleev University of Chemical Technology of Russia). Samples were weighed on a Vibra AJH-420 CE laboratory balance (Shinko Denshi Co., Ltd., Tokyo, Japan) with an accuracy of 0.0001 g. Zero-grade gaseous nitrogen (NII KM, Moscow, Russia) and liquid nitrogen [83] were used as the adsorbate. High-purity grade 5.5 helium (NII KM, Moscow, Russia) was used for volume calibration and free-space determination. Before measurement, each sample was vacuum-degassed at 150 °C and a residual pressure below 0.5 mmHg for 12 h to remove adsorbed moisture, residual volatiles, and gases from the surface and pores that could distort the measurements. The specific surface area was calculated using the Brunauer–Emmett–Teller (BET) equation; the micropore surface area was determined by the t-plot method; the micropore size distribution was calculated by the Horvath–Kawazoe method; and the meso- and macropore size distributions were calculated by the Barrett–Joyner–Halenda (BJH) method from the desorption branch of the isotherm (for BHB, for which no desorption branch was recorded, the adsorption branch was used). The total pore volume was obtained by summing the BJH incremental volumes.

### 4.5. Iodine Adsorption Analysis

The amounts of adsorbed iodine in aqueous solution were determined according to [84] without prior acid treatment of the samples. In brief, the biochar was dried at 110–115 °C in a drying oven to constant weight. Next, 1 g of the biochar sample was put in a conical flask with a ground-glass stopper and mixed with 100 mL of a 0.1 N iodine solution in potassium iodide. The resulting solution was mixed manually for 30 min. Subsequently, 10 mL of the supernatant solution was taken out and 0.1 mol/L of sodium thiosulfate standard solution was added to the supernatant solution in a dropwise manner. At the end of the titration, 1 mL of starch solution was added and the titration was continued until the blue color disappeared. The biochar’s iodine adsorption activity (X) as a percentage was calculated:

(1) $$X=\frac{\left(\;V_{1}-V_{2}\right)\cdot 12.7}{m}$$

where *V*_1_ is the volume of sodium thiosulfate solution with a concentration of 0.1 N spent on titration of 10 mL of iodine solution in potassium iodide in mL; *V*_2_ is the volume of sodium thiosulfate solution with a concentration of 0.1 N used for titration of 10 mL of iodine solution in potassium iodide, after treatment with biochar, in mL; *m* is the mass of the biochar sample to be tested in grams.

The result of the analysis is taken as the arithmetic mean of the results of two parallel determinations, the absolute discrepancy between which does not exceed the permissible discrepancy equal to 3%.

### 4.6. Analysis of Zearalenone Adsorption

The study examined the binding efficiency of ZEN at various pH levels (acidic—2.0 and alkaline—8.0), a standard temperature of 37 °C, and an incubation time of 120 min. Experiments were conducted at a toxin-to-sorbent mass ratio of 1:1000. This two-stage pH sequence (an acidic gastric phase followed by an alkaline intestinal phase) corresponds to established in vitro digestion models widely used to screen mycotoxin binders, which simulate the physiological pH shift from the stomach to the small intestine [19,74,85,86]. Analysis of the adsorption capacity of the sorbents involved the preparation of ZEN solutions. A stock ZEN solution with a concentration of 1 mg/mL was obtained by successively dissolving 1 mg of crystalline toxin (1.00 mg Sigma-Aldrich). The contents of the ampoule received from the manufacturer were transferred using alcohol [87] into a 1 mL volumetric lask, and the volume of the solution was brought to the mark with alcohol. Working alcohol solutions of ZEN were stored in a cool, dark place.

Sorbent samples (50 ± 0.5 mg) were weighed in sterile 15 mL polypropylene centrifuge tubes. 9.95 mL of a model solution with a pH 2 (0.9% saline sodium chloride (NaCl) adjusted to pH 2 with 0.1 N hydrochloric acid (HCl)) was added to the weighed sorbent samples in the tubes to simulate stomach conditions. Then, 0.05 mL of an alcohol solution containing the target mycotoxin (0.05 mg) was added. The final solution contained 0.5% alcohol. Alcohol serves as a carrier solvent for ZEN. The tubes were incubated at 37 °C for 120 min, shaking them manually periodically. After incubation, the solution was centrifuged for 15 min at 3000 rpm to obtain supernatants and sediment. Supernatants containing unbound ZEN were collected, filtered through a 0.22 µm membrane, and used for quantitative analysis. The pellet obtained from centrifugation was suspended in 10 mL of a buffered solution of sodium hydrogen phosphate (Na_2_HPO_4_ × 12H_2_O) (pH 8). The suspensions were incubated and centrifuged as described above, and the supernatants were collected. The adsorption efficiency was calculated as *A* = (*m*_0_ − *m*_1_)/*m*_0_ × 100%, where *m*_0_ = 0.05 mg is the mass of ZEN added and *m*_1_ is the mass of unbound ZEN in the supernatant after the pH 2.0 stage (measured concentration × 10 mL). Desorption was calculated as *D* = *m*_2_/(*m*_0_ − *m*_1_) × 100%, where *m*_2_ is the mass of ZEN released into the pH 8.0 buffer (measured con-centration × 10 mL), and the desorption-corrected efficiency as *AF* = *A* × (1 − *D*/100). The uptake at the tested dose was calculated as *q* = (*A*/100) × *m*_0_/*m*_s_, where *m*_s_ = 0.05 g is the sorbent mass.

ZEN in the supernatants was quantified by high-performance liquid chromatography–high-resolution tandem mass spectrometry (HPLC-HRMS/MS) using a Dionex UltiMate 3000 chromatograph (Thermo Fisher Scientific, San Jose, CA, USA) coupled with a Bruker Impact II mass spectrometer (Bruker Daltonics GmbH & Co. KG, Bremen, Germany). Before analysis, samples were filtered through 0.22 µm membrane filters to remove particulate matter. ZEN was identified and quantified by retention time, accurate mass of the molecular ion, and fragmentation spectrum. The calibration curve was constructed from a series of ZEN standard solutions (0.01–10 µg/mL) prepared by serial dilution with ethanol of a working solution (0.01 mg/mL) obtained from the ZEN stock solution (1 mg/mL; Sigma-Aldrich, purity ≥99%). The calibration was linear (*R*^2^ ≥ 0.999), and the limit of detection (LOD) was 0.005 µg/mL. Each analytical run included blanks (solvent without ZEN), a positive control (buffer with a known ZEN concentration without sorbent), and a negative control (buffer with sorbent without ZEN) to account for possible matrix effects and background mycotoxin levels [62].

### 4.7. General Toxicity

The method for determining general toxicity according to [88] consists of testing aqueous extracts and aqueous solutions of acetone extracts of the test samples. An average sample was ground in a laboratory mill and sieved through a 1 mm sieve. Two 10 g sample portions were weighed to prepare an aqueous solution of the acetone extract and an aqueous extract. To obtain the acetone extract, a 10 g sample was added to a specific amount of acetone, depending on the type of sample being tested, shaken, and allowed to settle. Then, 0.5 cm^3^ of the supernatant liquid from the extract was collected using an automatic pipette and transferred to a flask containing the Lozin-Lozinsky aqueous solution. To prepare the aqueous extract, a 10 g sample was added to 100 cm^3^ of distilled water, shaken for 20 min, filtered through a paper filter, and the supernatant was separated.

Biotesting was conducted using a 24-h test culture of the protozoan Stylonychia mytilus (ciliates). The culture medium was Lozin-Lozinsky’s aqueous solution, which contained sodium chloride, potassium chloride, calcium chloride, magnesium chloride, and sodium carbonate. The ciliates were fed fresh baker’s yeast, pre-dried at 55 °C for 2 days. Biotesting was performed using a well microaquarium unit. The 24-h ciliate culture was added using an automatic pipette, at 0.02 cm^3^ of the medium containing the ciliates to five microaquariums, along with 0.02 cm^3^ of an aqueous solution of acetone extract or 0.02 cm^3^ of an aqueous extract of the test sample prepared for biotesting. After 2 min, the ciliates in each well were counted. In accordance with [88], ciliate survival was recorded after 1, 2, 3, and 24 h of exposure to assess sample toxicity.

### 4.8. Statistics

All measurements were performed in triplicate, except for the iodine number (two parallel determinations) and the ciliate toxicity test (five replicate microaquariums per sample); results are presented as mean ± standard deviation. Differences between samples were evaluated by one-way analysis of variance (ANOVA) followed by Tukey’s (HSD) test; differences were considered statistically significant at *p* ≤ 0.05. No formal a priori sample-size (power) calculation was performed; triplicates were chosen in line with the design used in comparable in vitro mycotoxin-binding studies [62,89]. Pearson correlation coefficients were calculated from the mean values of the four samples (*n* = 4; two-tailed *t*-test, critical |*r*| = 0.950 at *p* < 0.05) and, given the small number of samples, were treated as descriptive.

### 4.9. Limitations and Scope

This work presents single-point adsorption efficiency data obtained under fixed experimental conditions (single ZEN dose: 0.05 mg per 50 mg biochar; pH 2 to 8; stage duration, 120 min; temperature, 37 °C). Full kinetic studies (e.g., pseudo-second-order models) and isotherms over a range of concentrations (e.g., Langmuir or Freundlich models) were beyond the scope of this study; the focus was on characterizing the physicochemical and textural properties of the biochars by FTIR, XRD, SEM, and low-temperature N_2_ adsorption (BET/BJH). We report the calculated single-point uptake (*q*, mg ZEN/g biochar) and removal efficiencies (%, *A* and *A_F_*), which allow comparison with other studies, and identify ZEN adsorption kinetics and isotherms as a direction for future work.

## 5. Conclusions

In this study, biochars were produced from four plant wastes differing in ash content and composition under identical pyrolysis conditions (550 °C, 10 °C/min, N_2_), and their in vitro zearalenone (ZEN)-binding ability was compared with their physicochemical properties to test the hypothesis that the mineral fraction plays a leading role.

Biochar yields were 30–41 wt%, with the highest solid yield obtained from rice husk. ACB and ARB contained 28–34 wt% ash dominated by K and Ca, with calcite and magnesian calcite as the main crystalline phases. RHB contained 44 wt% ash, consisting mainly of amorphous silica, whereas BHB was a low-ash (7 wt%), X-ray amorphous carbon material. All biochars had a very low specific surface area (0.45–2.30 m^2^/g) and negligible microporosity; their pore volume was formed by meso- and macropores. Iodine uptake (108–335 mg/g) was inconsistent with the very low N_2_-accessible surface area and therefore cannot be interpreted as a measure of microporosity. It likely reflects chemical interactions of iodine with the alkaline Ca/K-rich ash and reducing surface groups of the char.

Under conditions simulating the gastrointestinal tract, ZEN removal efficiency decreased in the order ACB > ARB > RHB > BHB (90.5 > 77.3 > 65.8 > 10.6%). Desorption at pH 8.0 did not exceed 1.5% of the bound toxin. All biochars were non-toxic in the *Stylonychia mytilus* test (survival > 70%), with the highest survival observed for ACB. Of the materials studied, ACB combines the highest ZEN removal efficiency, low desorption, and the highest ciliate survival. Its in vitro efficiency falls within the range reported for commercial sorbents, although direct comparison is limited by differences in protocols.

All three points of the hypothesis were satisfied at a qualitative level: the most carbonized, low-ash BHB was the least effective; biochars with carbonate Ca/K ash outperformed the biochar with siliceous ash; and RHB, which had the highest specific surface area, was inferior to the amaranth biochars. The amount of bound ZEN corresponded to approximately 45–96% of a monolayer on the N_2_-accessible surface; therefore, physical adsorption in these pores alone does not explain the differences between samples. At the same time, with four samples none of the correlations reached statistical significance, ash content, fixed carbon, and specific surface area co-varied, and the pH of the suspensions during the adsorption stage was not controlled. Therefore, the hypothesis that the mineral fraction plays an important role is only tentatively supported.

## Figures and Tables

**Table 1:** Results of the proximate analysis and mineral compositions of biochar.

	ACB	ARB	RHB	BHB
VM, wt%	24.60 ± 0.3	23.13 ± 0.6	12.39 ± 0.4	20.70 ± 0.6
Ash, wt%	27.65 ± 0.1	34.05 ± 0.5	43.53 ± 0.1	7.19 ± 0.03
FC *, wt%	47.75 ± 0.8	42.82 ± 0.5	44.08 ± 0.4	72.11 ± 0.9
Ca	35.70	33.47	0.70	19.05
K	49.83	47.25	2.59	33.75
Mg	4.08	2.57	0.30	6.83
P	3.87	2.60	0.34	n.d.
S	1.59	2.10	0.08	n.d.
Si	1.35	2.80	25.95	1.13
Cl	2.43	7.80	0.09	0.80
Fe	0.73	1.10	0.22	0.63
Mn	0.10	0.07	0.20	n.d.
Br	0.04	0.07	n.d.	n.d.
Sr	0.04	0.07	n.d.	n.d.
Zn	0.11	0.17	0.06	n.d.
Cu	n.d.	n.d.	0.01	n.d.
Ti	0.16	n.d.	n.d.	n.d.
Al	n.d.	n.d.	0.21	n.d.

* FC = 100 − VM − Ash. n.d.—not detected.

**Table 2:** Results of specific surface area determination.

Sample	S_BET_, m^2^/g	*R* ^2^	S_micro_ (t-plot), m^2^/g	S_micro_/S_BET_, %	V_BJH_, cm^3^/g	Mesopores (2–50 nm), %	Macropores (>50 nm), %
ACB	1.98 ± 0.01	0.99	0.161 ± 0.001	8.17 ± 0.08	0.0134	21.5	78.5
ARB	1.53 ± 0.01	0.99	0.381 ± 0.002	24.9 ± 0.3	0.0078	24.8	75.2
RHB	2.30 ± 0.05	0.99	0.209 ± 0.005	9.11 ± 0.41	0.0125	61.7	38.3
BHB	0.45 ± 0.02	0.99	n.d.	n.d.	0.0007 ^a^	0 ^a^	100 ^a^

S_BET_—BET specific surface area; S_micro_—micropore surface area; V_BJH_—cumulative volume of pores calculated as the sum of BJH incremental volumes (desorption branch; range 2.3–163 nm for ACB and ARB, 3.0–131 nm for RHB); n.d., not detected. ^a^ BJH adsorption branch (pore diameters 146–228 nm) because no desorption branch was detected; the value is at the detection limit of the method.

**Table 3:** The amounts of adsorbed iodine by biochar.

Sample	Pyrolysis Temperature (°C)	Heating Rate (°C/min)	Amounts (mg/g) *	Reference
ACB	550	10	291	This study
ARB	550	10	335	This study
RHB	550	10	145	This study
BHB	550	10	108	This study
biochar from sludge and peach wood	500	10	132	[51]
biochar from walnut shell	850	5	284	[52]

* (1% = 10 mg/g).

**Table 4:** In vitro adsorption properties of samples towards ZEN.

Name of the Adsorbent	In Vitro Adsorption of ZEN, %					Reference
	*A*	*D*	*A_F_*	*q*, mg/g	*q*(*A_F_*), mg/g	
ACB	90.5 ± 0.96	0.5 ± 0.08	90.05 ± 0.22	0.905	0.900	This study
ARB	77.3 ± 0.44	1.5 ± 0.09	76.14 ± 0.61	0.773	0.761	This study
RHB	65.8 ± 0.65	0.3 ± 0.00	65.61 ± 0.87	0.658	0.656	This study
BHB	10.6 ± 0.65	0	10.6 ± 0.65	0.106	0.106	This study
Activated coconut charcoal	97.6 ± 1.1	-	88.4 ± 1.2	-	-	[19]
Pine biochar	98.6 ± 1.2	-	86.7 ± 2.2	-	-	[19]
Horticulture biochar	46.0 ± 3.4	-	21.8 ± 4.3	-	-	[19]
Olive wood biochar	89.6 ± 2.3	-	55.4 ± 2.6	-	-	[19]
Avocado seed residues biochar	-	-	65.0 ± 5	-	-	[21]
Activated avocado seed residues biochar	-	-	70.0 ± 5	-	-	[21]
Nano-montmorillonite	-	-	95.0	-	-	[58]
Organokaolinite OKR-50	-	-	57.0	-	-	[59]
Benzalkonium modified zeolite BZ-2	-	-	38.0	-	-	[60]
Montmorillonite	-	-	49.0	-	-	[61]
Modified aluminosilicate *	52.0 ± 0.9	-	40.0 ± 2.2	-	-	[62]
Bentonite *	32.0 ± 0.4	-	29.0 ± 2.6	-	-	[62]
Activated clay *	44.0 ± 1.2	-	12.0 ± 1.2	-	-	[62]
Montmorillonite *	27.0 ± 2.3	-	18.0 ± 2.2	-	-	[62]
Shungite	86.3	0.5	85.8 ± 3.0	-	-	[63]
Lignin	84.4	3.2	81.2 ± 1.8	-	-	[63]
Zeolite	56.0	9.0	47.0 ± 2.2	-	-	[63]
Novasil Plus (BASF, Germany) *inorganic adsorbent*	79.0	-	77.61 ± 1.84	-	-	[64]
Escent^®^ S (Innovad^®^, Belgium) *complex adsorbent*	72.0	-	70.96 ± 0.75	-	-	[64]
Mycosorb^®^ (Alltech, USA) *organic adsorbent*	56 ± 2.2	-	53 ± 1.1	-	-	[62]

*A*—adsorption efficiency at pH 2.0; *D*—desorption at pH 8.0, % of bound ZEN; *A_F_* = *A* × (1 − *D*/100). * the trade name of the adsorbent is not indicated, but the name of the active substance is presented.

**Table 5:** Biochar toxicity on the survival of *Stylonychia mytilus*.

Sample	Survival in an Aqueous Solution of Acetone Extract (%)				Survival in Aqueous Extract (%)			
	1 h	2 h	3 h	24 h	1 h	2 h	3 h	24 h
ACB	99 ± 0.2	96 ± 0.2	96 ± 0.2	96 ± 0.2	98 ± 0.2	97 ± 0.2	97 ± 0.2	97 ± 0.2
ARB	77 ± 0.2	79 ± 0.2	79 ± 0.2	77 ± 0.2	97 ± 0.2	89 ± 0.2	89 ± 0.2	87 ± 0.2
RHB	92 ± 0.2	89 ± 0.2	86 ± 0.2	86 ± 0.2	97 ± 0.2	95 ± 0.2	95 ± 0.2	94 ± 0.2
BHB	91 ± 0.2	86 ± 0.2	84 ± 0.2	84 ± 0.2	95 ± 0.2	88 ± 0.2	88 ± 0.2	88 ± 0.2

**Table 6:** Input data for correlation analysis.

Sample	ZEN Adsorption, %	Ash, wt%	FC, wt%	VM, wt%	S_BET, m^2^/g	V_BJH, cm^3^/g	Dominant Ash Elements ^a^; Mineral Phase	ZEN Uptake per S_BET, µmol/m^2 b^
ACB	90.5	27.7	47.8	24.6	1.98	0.0134	K, Ca; calcite	1.44
ARB	77.3	34.1	42.8	23.1	1.53	0.0078	K, Ca; Mg-calcite	1.59
RHB	65.8	43.5	44.1	12.4	2.30	0.0125	Si; amorphous SiO_2_	0.90
BHB	10.6	7.2	72.1	20.7	0.45	0.0007	K, Ca	0.74

^a^ Elements ranked by their proportion in each sample. ^b^ *q*/(M_ZEN × S_BET), M_ZEN = 318.4 g/mol.

**Table 7:** Pearson correlation coefficients (*r*) of biochar properties with ZEN adsorption efficiency and BET surface area.

Parameter	*r* with ZEN Adsorption (*n* = 4)	*p*	*r* with ZEN Adsorption, Without BHB (*n* = 3)	*r* with S_BET_ (*n* = 4)
Ash	0.75	0.253	−0.99	0.92
FC	−0.91	0.086	0.74	−0.89
VM	0.20	0.800	0.90	−0.35
S_BET	0.84	0.160	−0.38	1.00
S_micro_	0.72	0.278	−0.25	0.55
V_BJH	0.89	0.109	0.19	0.97 *

S_micro_ for BHB was set to zero. For *n* = 4, *p* values were calculated from the *t*-distribution with 2 degrees of freedom (in this case, *p* = 1 − |*r*|); the critical |*r*| at *p* < 0.05 is 0.950. For *n* = 3, statistical significance cannot be established, and the coefficients are given for descriptive purposes only. * *p* < 0.05.

**Table 8:** Physicochemical properties of the initial biomass samples.

Characteristic	AC	AR	RH	BH
	Ultimate (wt%)—based on dry basis:			
Carbon	38.49 ± 0.1	36.97 ± 0.1	32.91 ± 0.1	39.33 ± 0.1
Hydrogen	6.09 ± 0.3	5.97 ± 0.1	4.15 ± 0.2	4.82 ± 0.2
Nitrogen	1.44 ± 0.5	4.49 ± 0.1	1.90 ± 0.2	2.09 ± 0.3
Sulfur	0.00	0.00	0.50 ± 0.01	0.30 ± 0.01
Oxygen *	41.00 ± 1.02	41.46 ± 0.2	42.02 ± 0.2	51.75 ± 0.2
	Proximate (wt%)—based on air-dried basis:			
Moisture	7.38 ± 0.03	5.75 ± 0.02	6.45 ± 0.03	6.90 ± 0.04
VM	75.80 ± 0.9	76.75 ± 1.7	66.60 ± 0.7	71.99 ± 0.8
Ash	12.98 ± 0.7	11.11 ± 0.5	18.52 ± 1.7	1.71 ± 0.05
FC	11.22 ± 0.2	12.14 ± 2.0	14.87 ± 2.2	26.29 ± 0.9

* calculated.

## Data Availability

The original contributions presented in this study are included in the article. Further inquiries can be directed to the corresponding author.

## References

[1] Kępińska-Pacelik J., Biel W. (2021). Mycotoxins—Prevention, Detection, Impact on Animal Health. Processes.

[2] Agriopoulou S., Stamatelopoulou E., Varzakas T. (2020). Advances in Occurrence, Importance, and Mycotoxin Control Strategies: Prevention and Detoxification in Foods. Foods.

[3] Lach M., Kotarska K. (2024). Negative Effects of Occurrence of Mycotoxins in Animal Feed and Biological Methods of Their Detoxification: A Review. Molecules.

[4] Xu R., Kiarie E.G., Yiannikouris A., Sun L., Karrow N.A. (2022). Nutritional impact of mycotoxins in food animal production and strategies for mitigation. J. Anim. Sci. Biotechnol..

[5] Deliklitas K., Gokbulut C. (2025). Prevention and detoxification of mycotoxins in food and feed: A review. Food Biosci..

[6] Ntungwe E.N., Tchana A.N., Abia W.A. (2024). Mycotoxin management: Exploring natural solutions for mycotoxin prevention and detoxification in food and feed. Mycotoxin Res..

[7] Fatullayeva S., Tagiyev D., Zeynalov N. (2021). A review on enterosorbents and their application in clinical practice: Removal of toxic metals. Colloid Interface Sci. Commun..

[8] Ropejko K., Twarużek M. (2021). Zearalenone and Its Metabolites—General Overview, Occurrence, and Toxicity. Toxins.

[9] Han X., Huangfu B., Xu T., Xu W., Asakiya C., Huang K., He X. (2022). Research Progress of Safety of Zearalenone: A Review. Toxins.

[10] Chilaka C.A., De Boevre M., Atanda O.O., De Saeger S. (2018). Stability of fumonisin B 1, deoxynivalenol, zearalenone, and T-2 toxin during processing of traditional Nigerian beer and spices. Mycotoxin Res..

[11] Sadok I., Krzyszczak-Turczyn A., Czech B., Parlakidis P., Vryzas Z. (2025). Advancements in biochar-based materials for decontamination and analytical detection of pesticides and mycotoxins in food. Food Chem..

[12] Enaime G., Wichern M., Lübken M. (2023). Contribution of biochar application to the promotion of circular economy in agriculture. Front. Agron..

[13] Kazemi M. (2025). Biochar in Animal Agriculture: Enhancing Health, Efficiency and Environmental Sustainability. Vet. Med. Sci..

[14] Rajput V., Saini I., Parmar S., Pundir V., Kumar V., Kumar V., Naik B., Rustagi S. (2024). Biochar production methods and their transformative potential for environmental remediation. Discov. Appl. Sci..

[15] Dayoub E.B., Tóth Z., Soós G., Anda A. (2024). Chemical and Physical Properties of Selected Biochar Types and a Few Application Methods in Agriculture. Agronomy.

[16] Tomczyk A., Sokołowska Z., Boguta P. (2020). Biochar physicochemical properties: Pyrolysis temperature and feedstock kind effects. Rev. Environ. Sci. Biotechnol..

[17] Ogunwa K.I., Nnadozie E.C., Dube N., Oladoye P.O., Obayomi K.S. (2026). The evolving role of biochar: Recent advances and future directions—A review. Biomass Bioenergy.

[18] Raz M., Bagherzadeh-Kasmani F., Karimi-Torshizi M.A., Ghazaghi M., Mokhtarpour A., Mehri M. (2025). *Prosopis farcta* biochar neutralizes aflatoxin B1 and enhances health and productivity in quails. Discov. Anim..

[19] Appell M., Wegener E.C., Sharma B.K., Eller F.J., Evans K.O., Compton D.L. (2023). In Vitro Evaluation of the Adsorption Efficacy of Biochar Materials on Aflatoxin B1, Ochratoxin A, and Zearalenone. Animals.

[20] Han J., Zhang J., Meng J., Cai Y., Cheng M., Wu S., Li Z. (2023). Characterization of modified rice straw biochar in immobilizing *Bacillus subtilis* 168 and evaluation on its role as a novel agent for zearalenone-removal delivery. J. Hazard. Mater..

[21] Gomez-Vilchis J.C., García-Rosales G., Longoria-Gandara L.C., Pérez-Gomez E.O., Tenorio-Castilleros D. (2025). Maximizing zearalenone removal: Unveiling the superior efficiency of pre-treated carbon in adsorption and photocatalysis. Heliyon.

[22] Bueno D.J., Di Marco L., Oliver G., Bardón A. (2005). In vitro binding of zearalenone to different adsorbents. J. Food Prot..

[23] Karami S., Papari S., Klinghoffer N.B., Berruti F. (2022). Pyrolytic Conversion of Vomitoxin-Contaminated Corn into Value-Added Products. Sustainability.

[24] Nair P.S., Sivani Menonm P.S., Suresh S., Sreekanth A.J., Sivasabari K., Adithya Krishna S., Anuranj P.R., Krishnan N., Parvathy S., Chakraborty S. (2023). Beneficial impacts of biochar as a potential feed additive in animal husbandry. J. Exp. Biol. Agric. Sci..

[25] Jindo K., Mizumoto H., Sawada Y., Sanchez-Monedero M.A., Sonoki T. (2014). Physical and chemical characterization of biochars derived from different agricultural residues. Biogeosciences.

[26] Ćwieląg-Piasecka I., Jamroz E., Medyńska-Juraszek A., Bednik M., Kosyk B., Polláková N. (2023). Deashed Wheat-Straw Biochar as a Potential Superabsorbent for Pesticides. Materials.

[27] Karaeva J.V., Timofeeva S.S., Islamova S.I., Slobozhaninova M.V., Bolour E. (2025). Prospects of Processing Buckwheat Husk into Biochar. Ecol. Ind. Russ..

[28] Collard F.-X., Blin J. (2014). A review on pyrolysis of biomass constituents: Mechanisms and composition of the products obtained from the conversion of cellulose, hemicelluloses and lignin. Renew. Sustain. Energy Rev..

[29] Imam T., Capareda S. (2012). Characterization of bio-oil, syn-gas and bio-char from switchgrass pyrolysis at various temperatures. J. Anal. Appl. Pyrolysis.

[30] de Meira A.M., Nolasco A.M., Klingenberg D., de Souza E.C., Dias Júnior A.F. (2021). Insights into the reuse of urban forestry wood waste for charcoal production. Clean Technol. Environ. Policy.

[31] Bhattacharjee N., Biswas A.B. (2018). Pyrolysis of *Alternanthera philoxeroides* (alligator weed): Effect of pyrolysis parameter on product yield and characterization of liquid product and bio char. J. Energy Inst..

[32] Taib R.M., Abdullah N., Aziz N.S.M. (2021). Bio-oil derived from banana pseudo-stem via fast pyrolysis process. Biomass Bioenergy.

[33] Kluska J., Matej-Łukowicz K., Nawrot N. (2024). Characterization of sunflower waste carbonization: Energy balance and water holding properties. Sustainability.

[34] Vuppaladadiyam A.K., Vuppaladadiyam S.S.V., Sahoo A., Murugavelh S., Anthony E., Bhaskar T., Zheng Y., Zhao M., Duan H., Zhao Y. (2023). Bio-oil and biochar from the pyrolytic conversion of biomass: A current and future perspective on the trade-off between economic, environmental, and technical indicators. Sci. Total Environ..

[35] Akca M.O., Ok S.S., Deniz K., Mohammedelnour A., Kibar M. (2021). Spectroscopic characterisation and elemental composition of biochars obtained from different agricultural wastes. J. Agric. Sci..

[36] Armynah B., Atika, Djafar Z., Piarah W.H., Tahir D. (2018). Analysis of Chemical and Physical Properties of Biochar from Rice Husk Biomass. J. Phys. Conf. Ser..

[37] Faizal A.N.M., Wen C.H., Putra N.R., Zaini A.S., Agi A., Nordin A.H., Zaini M.A.A. (2025). Cray-fish shell biochar for methyl violet adsorption: Equilibrium and kinetic studies. Next Sustain..

[38] Hossain N., Nizamuddin S., Griffin G., Selvakannan P., Mubarak N.M., Mahlia T.M.I. (2020). Synthesis and characterization of rice husk biochar via hydrothermal carbonization for wastewater treatment and biofuel production. Sci. Rep..

[39] Pradhani N.S., Mulyanto D. (2025). Characterization of Functional Groups in Sengon Biochar Under Various Pyrolysis Temperatures. Soil Water J..

[40] Janu R., Malik U., Ribitsch D., Hofman J., Sedláček P., Bielská L., Soja G. (2021). Biochar Surface Functional Groups as Affected by Biomass Feedstock, Biochar Composition and Pyrolysis Temperature. Carbon Resour. Convers..

[41] Officerov E.N., Korosteleva Y.A. (2005). The use of Amaranth in solving the problem of hypocalcemia. Butlerov Commun..

[42] Popova N.F., Kuchaeva L.N., Osmolovskaya N.G. (2007). Effect of mineral nutrition conditions on the formation of oxalate pools in the leaves of *Amaranthus cruentus*. Bull. St. Petersburg Univ..

[43] Lee S.H., Kang D.S., Lee S.M., Roh J.S. (2018). X-ray diffraction analysis of the effect of ball milling time on crystallinity of milled polyacrylonitrile-based carbon fiber. Carbon Lett..

[44] Dias J., Conceição A., Delatorre F.M., Siqueira P. (2025). Production, Characterization Physical, Chemical, and Structural Analysis of Biochar Fines for Bio-Reinforcement in Composite Materials. Processes.

[45] Severo F.F., da Silva L.S., Moscôso J.S.C., Sarfaraz Q., Júnior L.F.R., Lopes A.F., Marzari L.B., Molin G.D. (2020). Chemical and physical characterization of rice husk biochar and ashes and their iron adsorption capacity. Appl. Sci..

[46] Thommes M., Kaneko K., Neimark A.V., Olivier J.P., Rodriguez-Reinoso F., Rouquerol J., Sing K.S.W. (2015). Physisorption of gases, with special reference to the evaluation of surface area and pore size distribution (IUPAC Technical Report). Pure Appl. Chem..

[47] Tsai W.-T., Lin Y.-Q., Huang H.-J. (2021). Valorization of Rice Husk for the Production of Porous Biochar Materials. Fermentation.

[48] Zama E.F., Zhu Y.-G., Reid B.J., Sun G.-X. (2017). The role of biochar properties in influencing the sorption and desorption of Pb(II), Cd(II) and As(III) in aqueous solution. J. Clean. Prod..

[49] Demir Z., Acar Bozkurt P. (2026). Co-pyrolysis of agricultural biomass for potentially functional biochar: Combined influence of both feedstocks and structural characterization. Sci. Rep..

[50] Sigmund G., Hüffer T., Hofmann T., Kah M. (2017). Biochar total surface area and total pore volume determined by N_2_ and CO_2_ physisorption are strongly influenced by degassing temperature. Sci. Total Environ..

[51] Bai B., Liu Q., Li H., Liu D., Wang H., Zhang C., Yang Z., Yao J. (2024). Sorption of Iodine on Biochar Derived from the Processing of Urban Sludge and Garden Waste at Different Pyrolysis Temperatures. Molecules.

[52] Sabitov A., Turganbay S., Kerimkulova A., Doszhanov Y., Saurykova K., Atamanov M., Zhumazhanov A., Bolatova D. (2025). 1-Carboxy-2-phenylethan-1-aminium Iodide 2-Azaniumyl-3-phenylpropanoate Crystals: Properties and Its Biochar-Based Application for Iodine Enrichment of Parsley. Appl. Sci..

[53] Mahmoud E.R.I., Aly H.M., Hassan N.A., Aljabri A., Khan A.L., El-Labban H.F. (2024). Utilizing Date Palm Leaf Biochar for Simultaneous Adsorption of Pb(II) and Iodine from Aqueous Solutions. Processes.

[54] Hernandez-Maglinao J., Capareda S.C. (2019). Improving the Surface Areas and Pore Volumes of Biochar Produced from Pyrolysis of Cotton Gin Trash via Steam Activation Process. Int. J. Eng. Sci..

[55] Castiglioni M., Rivoira L., Ingrando I., Del Bubba M., Bruzzoniti M.C. (2021). Characterization Techniques as Supporting Tools for the Interpretation of Biochar Adsorption Efficiency in Water Treatment: A Critical Review. Molecules.

[56] (2021). Standard Test Method for Determination of Iodine Number of Activated Carbon.

[57] Zhang Y., Xu X., Cao L., Ok Y.S., Cao X. (2018). Characterization and quantification of electron donating capacity and its structure dependence in biochar derived from three waste biomasses. Chemosphere.

[58] Zhang W., Zhang L., Jiang X., Liu X., Li Y., Zhang Y. (2020). Enhanced adsorption removal of aflatoxin B1, zearalenone and deoxynivalenol from dairy cow rumen fluid by modified nano-montmorillonite and evaluation of its mechanism. Anim. Feed Sci. Technol..

[59] Spasojević M., Daković A., Rottinghaus G.E., Obradović M., Krajišnik D., Marković M., Krstić J. (2021). Influence of surface coverage of kaolin with surfactant ions on adsorption of ochratoxin A and zearalenone. Appl. Clay Sci..

[60] Marković M., Daković A., Rottinghaus G.E., Petković A., Kragović M., Krajišnik D., Milić J. (2017). Ochratoxin A and zearalenone adsorption by the natural zeolite treated with benzalkonium chloride. Colloids Surf. A Physicochem. Eng. Asp..

[61] Bekci Z.M., Antep M.K., Merdivan M., Yurdakoç K. (2011). Zearalenone removal in synthetic media and aqueous part of canned corn by montmorillonite K10 and pillared montmorillonite K10. J. Food Prot..

[62] Kolawole O., Meneely J., Greer B., Chevallier O., Jones D.S., Connolly L., Elliott C. (2019). Comparative In Vitro Assessment of a Range of Commercial Feed Additives with Multiple Mycotoxin Binding Claims. Toxins.

[63] Mishina N.N., Semenov E.I., Perfilova K.V., Yamalova G.R., Aleev D.V. (2023). The ability of heterogeneous sorbents to the binding of Zearalenone in vitro. Bull. Kursk. State Agric. Acad..

[64] Tarasova E.Y., Matrosova L.E., Sadykova A.S. (2021). Efficiency of Zearalenon adsorption in vitro. Issues Leg. Regul. Vet. Med..

[65] Wang L., Chen Y., Zhao Y., Du M., Wang Y., Fan J., Ren N., Lee D.-J. (2020). Toxicity of two tetracycline anti-biotics on Stentor coeruleus and *Stylonychia lemnae*: Potential use as toxicity indicator. Chemosphere.

[66] Antil S., Varatharajan G.R., Ndayishimiye J.C., Nyirabuhoro P., Saldaev D., Kumar S., Toteja R., Makhija S., Terza A.L., Mazei Y.A. (2026). Ciliates as next-generation bioindicators in pollution-impacted freshwaters: Integrating community signatures with stress-response endpoints. J. Microbiol. Methods.

[67] Tyan E.A., Permyakov A.A., Kotomina G.A. (2018). Determination of general toxicity of oatmeals «hercules» by biotasting at (*Stylonychia mytilus*) infusions. Innov. Food Saf..

[68] Kwiatkowski M., Kalderis D. (2020). A complementary analysis of the porous structure of biochars obtained from biomass. Carbon Lett..

[69] Agapkin A., Makhotina I., Karagodin V., Utkina A., Kalachev S., Izembayeva A. (2023). Agricultural waste processing technology and its relationship with the organic products industry. Bulg. J. Agric. Sci..

[70] Karaeva J., Timofeeva S., Islamova S., Slobozhaninova M., Oleynikova E., Sidorkina O. (2025). High-Value Utilization of Amaranth Residue and Waste LDPE by Co-Pyrolysis. Molecules.

[71] Karaeva J., Timofeeva S., Islamova S., Bulygina K., Aliev F., Panchenko V., Bolshev V. (2023). Pyrolysis of Amaranth Inflorescence Wastes: Bioenergy Potential, Biochar and Hydrocarbon Rich Bio-Oil Production. Agriculture.

[72] Sorokin A.V., Skorlukhanova A.V., Tsimmer E.M., Lavlinskaya M.S. (2025). Exploring the use of rice husk processing byproducts in composite superabsorbent material synthesis. Chem. Plant Raw Mater..

[73] Kordi M., Farrokhi N., Pech-Canul M.I., Ahmadikhah A. (2024). Rice Husk at a Glance: From Agro-Industrial to Modern Applications. Rice Sci..

[74] Kihal A., Rodríguez-Prado M., Calsamiglia S. (2022). The efficacy of mycotoxin binders to control mycotoxins in feeds and the potential risk of interactions with nutrient: A review. J. Anim. Sci..

[75] Tomašević-Čanović M., Daković A., Rottinghaus G., Matijašević S., Đuričić M. (2003). Surfactant modified zeolites—New efficient adsorbents for mycotoxins. Microporous Mesoporous Mater..

[76] Yang X., Yao M., Liao W., Li X. (2025). Adsorption–Degradation Integrated Approaches to Mycotoxin Removal from Food Matrices: A Comprehensive Review. Toxins.

[77] Danat B.T., Wuana R.A., Chahul H.F. (2026). Review of adsorption isotherms models. Appl. Water Sci..

[78] (2020). Standard Test Method for Ash in Biomass.

[79] (2020). Standard Test Method for Determination of Total Solids in Biomass.

[80] (2016). Determination of Ash Content by the Standard Method.

[81] (2015). Solid Biofuel. Determination of Volatile Matter Content.

[82] Karaeva J.V., Timofeeva S.S., Islamova S.I., Gerasimov A.V. (2022). Pyrolysis kinetics of new bioenergy feedstock from anaerobic digestate of agro-waste by thermogravimetric analysis. J. Environ. Chem. Eng..

[83] (2007). Gaseous and Liquid Nitrogen. Technical Specifications.

[84] (2003). Crushed activated wood Charcoal.

[85] Boisen S., Fernandez J.A. (1997). Prediction of the total tract digestibility of energy in feedstuffs and pig diets by in vitro analyses. Anim. Feed Sci. Technol..

[86] Kong C., Shin S.Y., Kim B.G. (2014). Evaluation of mycotoxin sequestering agents for af-latoxin and deoxynivalenol: An in vitro approach. SpringerPlus.

[87] (2014). Rectified Ethyl Alcohol Derived from Food-Grade Raw Materials.

[88] (2014). Animal Feed, Compound Feed, Raw Materials for Compound Feed.

[89] Nesic K., Jaksic S., Popov N., Zivkov-Balos M., Pajic M., Zloh B., Polacek V. (2020). In Vitro assessment of binding capacity of combined adsorbent (bentonite with yeast cell wall ex-tracts) and Aflatoxin B1. Arch. Vet. Med..

